# Constructing Mn‐Co‐Fe Ternary Metal Phosphides Nanosheet Arrays as Bifunctional Electrocatalysts for Overall Water Splitting

**DOI:** 10.1002/advs.202417521

**Published:** 2025-03-27

**Authors:** Fan Wang, Zhen Pei, Zhou Xu, Tengteng Qin, Xin Ouyang, Dongyun Li, Yang Hou, Xingzhong Guo

**Affiliations:** ^1^ State Key Laboratory of Silicon and Advanced Semiconductor Materials School of Materials Science and Engineering Zhejiang University Hangzhou 310058 China; ^2^ College of Materials and Chemistry China Jiliang University Hangzhou 310018 China; ^3^ Key Laboratory of Biomass Chemical Engineering of Ministry of Education College of Chemical and Biological Engineering Zhejiang University Hangzhou 310027 China; ^4^ Hangzhou Global Scientific and Technological Innovation Center Zhejiang University Hangzhou 311200 China

**Keywords:** bifunctional electrocatalysts, heterostructure, overall water splitting, transition metal phosphides

## Abstract

Bifunctional electrocatalysts with high efficiency, stability, and distinguished performance have attracted more and more attention in the field of overall water splitting, while the composition and structure design are very essential for electrocatalysts with superb performance and low price. In this work, the heterostructure Mn‐Co‐Fe‐P nanoarrays is in situ grown on nickel foam (NF) by simple hydrothermal method and phosphating method. The resultant Mn‐Co‐Fe‐P catalyst has favorable electrocatalytic performance with the oxygen evolution reaction (OER) overpotential at 10 (100) mA cm^−2^ of 192 (279) mV and the Tafel slope of 43.75 mV dec^−1^, the hydrogen evolution reaction (HER) overpotential at 10 (100) mA cm^−2^ of 98 (152) mV and the Tafel slope of 40.68 mV dec^−1^, and the full voltage of 1.66 V at 100 mA cm^−2^ when applied to overall water splitting. The 3D heterostructure provides more active sites, and the in situ growth improves the stability and conductivity of the catalyst. This binder‐free heterostructure electrocatalyst with excellent stability and catalytic performance is a promising bifunctional electrocatalyst candidate for overall water splitting.

## Graphical abstract

The heterostructure Mn‐Co‐Fe‐P nanoarrays is successfully fabricated on self‐supporting nickel foam by a facile hydrothermal method and phosphating method. In situ growth improves the stability and conductivity of Mn‐Co‐Fe‐P electrocatalysts, and the 3D “sheet–sheet” structure expands the specific surface area of the catalyst and exposes more active sites, endowing Mn‐Co‐Fe‐P outstanding electrocatalytic properties.

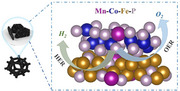

## 1 Introduction

The energy crisis has forced people to look for clean and renewable alternative energy sources, among which hydrogen energy has attracted widespread attention. Hydrogen has the potential to become one of the future energy sources due to its high energy density, cleanness, and regeneration. Among hydrogen production technologies, overall water splitting for hydrogen production is one of the measures to get new energy.^[^ ^1^, ^2^, ^3^, ^4^ ^]^ In theory, the minimum voltage required for water electrolysis is 1.23 V, while the complex process of hydrogen production from water electrolysis greatly increases the voltage required for water electrolysis.^[^ ^5^, ^6^ ^]^ Industrially efficient electrocatalysts are generally noble metal‐based electrocatalysts such as Pt‐based and Ir‐based electrocatalysts. However, these precious metals are extremely rare in nature and costly, which largely limits their extensive application in the field of electrocatalysis.^[^ ^7^, ^8^ ^]^ Therefore, alternative catalysts, such as metal carbides, metal hydroxides, metal sulfide, and metal phosphides, especially transition metal phosphides, have been developed to replace these expensive electrocatalysts.^[^ ^9^, ^10^ ^]^

Transition metal phosphides have good electrical conductivity and remarkable catalytic performance and are widely used as HER and OER catalysts.^[^ ^11^ ^]^ Cobalt phosphide and iron phosphide are often used in water electrolysis because of their rich content and distinguished catalytic activity.^[^ ^12^, ^13^, ^14^ ^]^ Jiang et al.^[^ ^15^ ^]^ reported Fe‐doped CoP nanocube/CoP nanosheet array heterostructure (CoFeP/CoP/CC) electrocatalysts on carbon cloth. The synthesized CoFeP/CoP/CC catalysts have abundant heterogeneous interfaces, which expose more reactive sites and regulate the electronic structure. In addition, the strong interfacial coupling of CoFeP to CoP and the integrated structure on carbon cloth ensure high electronic conductivity and enhanced mechanical stability. Benefiting from these advantages, CoFeP/CoP/CC exhibits significant OER performance with an overpotential of 240 mV at a current density of 10 mA cm^−2^ and remarkable OER catalytic stability within 100 h. Kumar et al.^[^ ^16^ ^]^ reported a rod‐like 1D non‐precious metal FeCoP catalyst by a “one‐pot” colloidal method, which exhibited outstanding OER activity with a current density of up to 950 mA cm^−2^ and a Tafel slope of 54 mV dec^−1^. The OER overpotentials of the FeCoP electrocatalyst are 230 and 260 mV at current densities of 50 and 100 mA cm^−2^ in 1.0 m KOH, respectively. The results show that a large number of electrocatalytic active sites enhance the OER kinetics process by providing metal ion sites to facilitate in situ surface formation and adsorption of ^*^O, ^*^OH, ^*^OOH reactive species. In the meantime, the structural design of the catalyst has a great influence on the catalytic performance. The rational design of the 3D structure can effectively increase the specific surface area and accelerate the material transfer rate.^[^ ^17^, ^18^ ^]^ Precisely controlling the morphology and structure of the catalyst can availably improve the electrocatalytic performance.^[^ ^19^, ^20^ ^]^

Herein, we demonstrate the construction of heterostructure Mn‐Co‐Fe‐P nanoarrays bifunctional catalyst for water electrolysis by a facile two‐step hydrothermal method and phosphating. The morphology‐controllable “sheet–sheet” heterostructure and the unique structure with more catalytic active sites and fast material transport channels are constructed for Mn‐Co‐Fe‐P electrocatalysts. Simultaneously, the synergistic effect of the heterostructure optimizes the electronic structure inside the catalyst and improves the conductivity and electron transfer rate. The heterostructure Mn‐Co‐Fe‐P nanoarrays electrocatalyst has the advantages of low overpotential, brilliant catalytic performance, and admirable stability in alkaline electrolytes, illustrating that the heterostructure Mn‐Co‐Fe‐P nanoarrays bifunctional electrocatalyst has a capacious application prospect.

## 2 Results and Discussion

### 2.1 Characteristics of the Heterostructure Mn‐Co‐Fe‐P Nanoarrays

**Figure 1:**
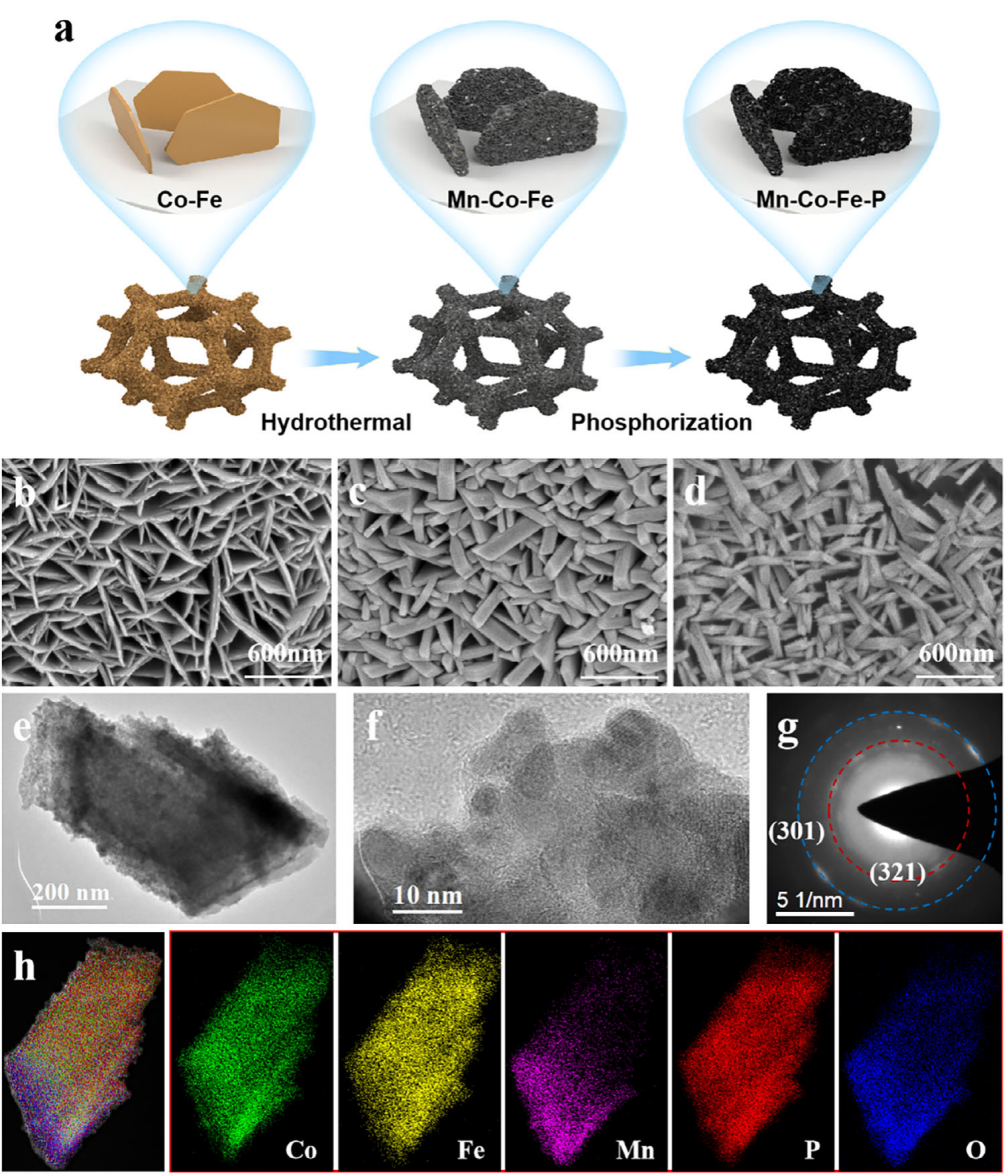
a) The synthesis process of the heterostructure Mn‐Co‐Fe‐P nanoarrays; FESEM images of b) Co–Fe precursor, c) Mn‐Co‐Fe precursor and d) Mn‐Co‐Fe‐P; e) TEM, f) HRTEM, g) selected area electron diffraction (SAED) and h) elemental mapping images of Mn‐Co‐Fe‐P.

The synthesis procedure of the heterostructure Mn‐Co‐Fe‐P nanoarrays is illustrated in **Figure** **1**a. First, the Co–Fe precursor is grown on nickel foam by hydrothermal method, and then Mn‐Co‐Fe precursor is obtained by the secondary hydrothermal method. Finally, the “sheet–sheet” heterostructure Mn‐Co‐Fe‐P is acquired by phosphating treatment.

Field emission scanning electron microscopy (FESEM) images (Figure 1b; Figure S1a, Supporting Information) show that the Co‐Fe precursor nanosheets grow vertically on the surface of nickel foam with a thickness of ≈60–70 nm. After secondary hydrothermal treatment, high‐valent Mn ions are reduced to low‐valent Mn ions during the hydrothermal redox process. After interaction with Co–Fe precursor, uniform and ultra‐thin small nanosheets are formed on the surface of nanosheets, obtaining a heterostructure Mn‐Co‐Fe precursor, as shown in Figure 1c and Figure S1b (Supporting Information). After phosphating, the precursor is thermally decomposed and gaseous water escapes to form a porous wall structure. The morphology of 3D heterostructure Mn‐Co‐Fe‐P nanoarrays remains intact. The 3D heterostructure structure formed by the secondary hydrothermal method and phosphating method further expands the specific surface area of the catalyst, increases the active sites, and contact area between the catalyst and the electrolyte, and further improves the catalytic performance. It can be seen from Figure 1e that Mn‐Co‐Fe‐P is composed of fine nanosheets. The lattice spacing measured by high‐resolution transmission electron microscopy (HRTEM) in Figure 1f and Figure S2 (Supporting Information) are 0.188 and 0.199 nm, corresponding to the (202) and (141) crystal planes of CoP and Fe_3_P, respectively. The selected‐area electron diffraction collected on the Mn‐Co‐Fe‐P nanoarray is shown in Figure 1g, which is well matched to the (301) and (321) crystal planes of CoP and Fe_3_P, respectively. Furthermore, the elemental distribution in Figure 1h demonstrates that Co, Fe, Mn, P, and O are uniformly distributed in Mn‐Co‐Fe‐P, confirming the existence of the above elements.

In addition, the influence of Mn contents and cobalt‐iron ratios on the morphology of Mn‐Co‐Fe‐P are also studied, as shown in Figure S3 (Supporting Information). From Figure S3a,b (Supporting Information), it can be seen that the concentration of KMnO_4_ solution has no obvious effect on the microstructure of the catalyst. If the concentration of KMnO_4_ is reduced or increased, it can be observed that the morphology of the catalyst remains unchanged, still maintaining the “sheet–sheet” shape structure. However, it can be seen from Figure S3c,d (Supporting Information) that different additions of Co(NO_3_)_2_·6H_2_O and Fe(NO_3_)_3_·9H_2_O will form nanoarrays with different morphologies. When the addition amount of Co(NO_3_)_2_·6H_2_O increases to 0.75 mmol, while the addition amount of Fe(NO_3_)_3_·9H_2_O reduces to 0.25 mmol, the formed heterostructure is a “wire‐sheet” array. The first step of hydrothermal reaction form nanowire arrays on the nickel foam. With the reduction of cobalt precursors and the increase of iron precursors, the first hydrothermal reaction will form nanosheet arrays, and finally a “sheet–sheet” heterostructure is prepared. Moreover, the MnCoFe‐P obtained by the one‐step hydrothermal process is some block material (Figure S4a, Supporting Information).

**Figure 2:**
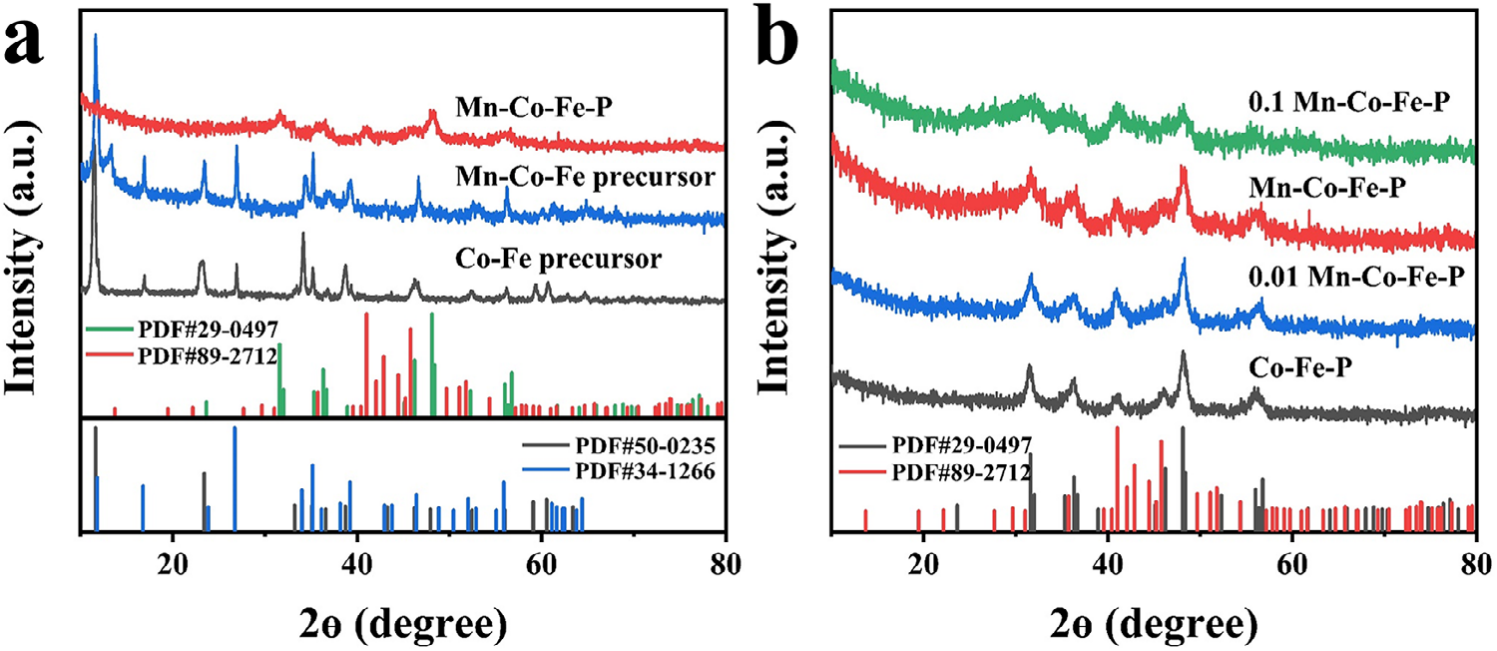
XRD patterns of as‐prepared a) Co–Fe precursor, Mn‐Co‐Fe precursor, Mn‐Co‐Fe‐P and b) Co‐Fe‐P, 0.01 Mn‐Co‐Fe‐P, Mn‐Co‐Fe‐P, 0.1 Mn‐Co‐Fe‐P.

The phase composition of the samples is analyzed by X‐ray diffraction (XRD). **Figure** **2**a shows the XRD patterns of Co–Fe precursor, Mn‐Co‐Fe precursor, and Mn‐Co‐Fe‐P. It can be seen that the peaks located at 11.6° and 23.4° correspond to (003) and (006) crystal planes of Co_5.84_Fe_2.16_(OH)_16_(CO_3_)_1.08_·0.32H_2_O (PDF#50‐0235), the peaks at 16.8°, 26.7°, 35.2°, 39.2°, 46.4°, and 55.9° could be indexed to (200), (310), (211), (301), (411), and (521) crystal planes of FeO(OH) (PDF#34‐1266), conforming the formation of Co–Fe hydroxide nanosheets. After phosphating heat treatment, in the Mn‐Co‐Fe‐P, the diffraction peaks at 31.6°, 36.3°, 48.2°, and 56.8° correspond to (011), (111), (211), and (301) crystal planes of CoP (PDF#29‐0497), and the diffraction peaks at 41.1° and 45.8° correspond to the (321) and (141) crystal planes of Fe_3_P (PDF#89‐2712). Figure 2b shows Co‐Fe‐P and Mn‐Co‐Fe‐P samples under different Mn concentrations. It can be seen that the XRD pattern of Mn‐Co‐Fe‐P is the same as that of Co‐Fe‐P, while no characteristic peaks of Mn are observed in the Mn‐Co‐Fe precursor and Mn‐Co‐Fe‐P in Figure 2a, indicating that Mn should exist in a doped form.^[^ ^21^ ^]^ According to Figure S4b (Supporting Information), MnCoFe‐P is composed of CoP and Fe_3_P. Table S1 (Supporting Information) is the element content of Mn, Co, Fe, and P in the Mn‐Co‐Fe‐P sample measured by inductively coupled plasma‐mass spectrometry (ICP‐MS), from which it can be seen that the atomic ratio of Co and Fe is ≈1.

**Figure 3:**
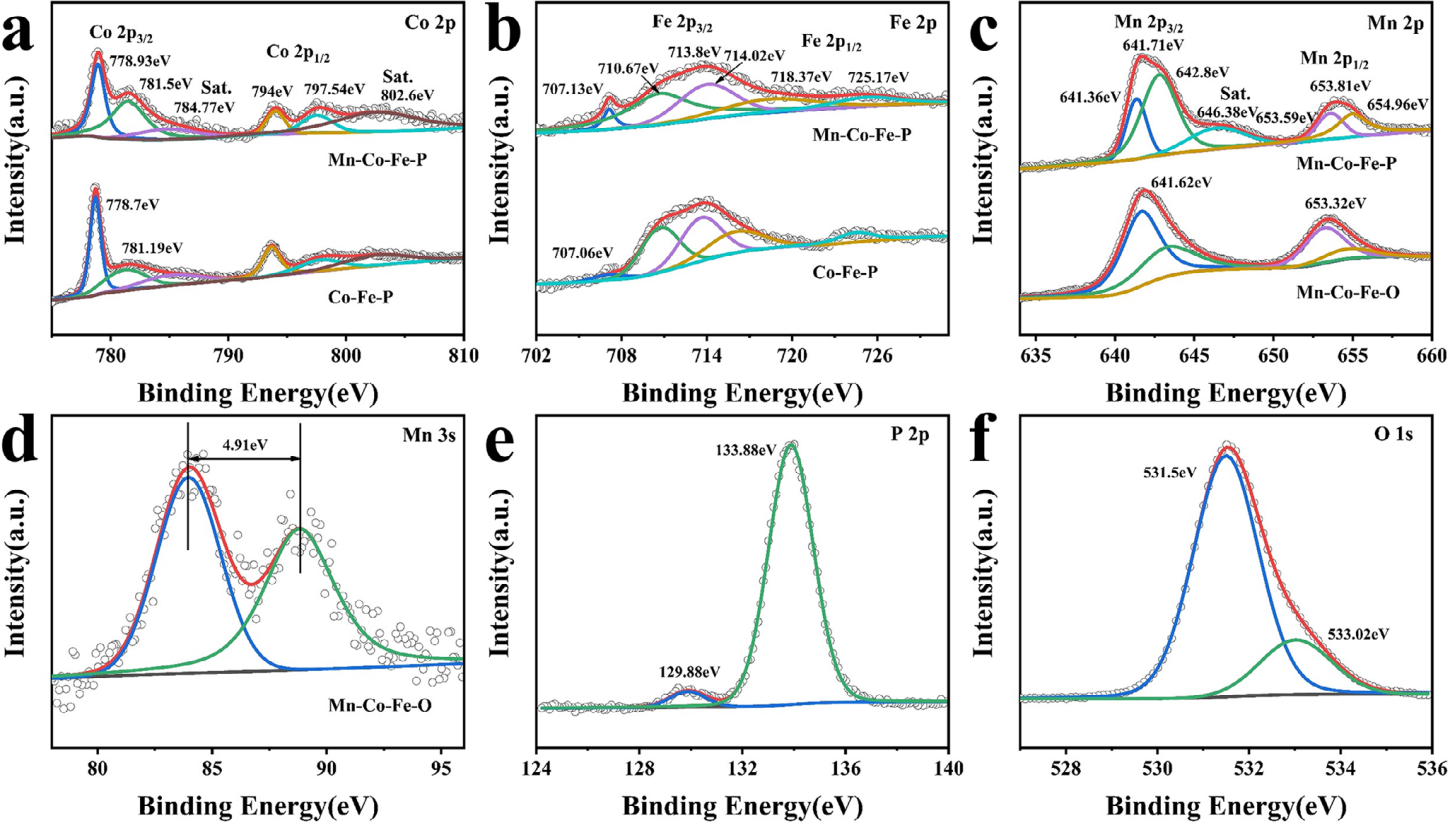
XPS spectra of Mn‐Co‐Fe‐P, Co‐Fe‐P, and Mn‐Co‐Fe‐O: a) Co 2p of Mn‐Co‐Fe‐P and Co‐Fe‐P, b) Fe 2p of Mn‐Co‐Fe‐P and Co‐Fe‐P, c) Mn 2p of Mn‐Co‐Fe‐P and Mn‐Co‐Fe‐O, d) Mn 3s of Mn‐Co‐Fe‐O, e) P 2p and f) O 1s of Mn‐Co‐Fe‐P.

The elemental composition and chemical valence state of the samples are analyzed by X‐ray photoelectron spectroscopy (XPS), and the results are displayed in **Figure** **3** and Figure S5 (Supporting Information). The full XPS spectra of Co‐Fe‐P, Mn‐Co‐Fe‐O, and Mn‐Co‐Fe‐P prove the existence of Mn, Co, Fe, P, and O elements. In the Co 2p spectrum of Mn‐Co‐Fe‐P, the peaks at 778.93, 781.5, and 784.77 eV correspond to Co–P, the oxidation state of Co, and the satellite peak at Co 2p_3/2_, respectively. The three peaks at 794, 797.54, and 802.6 eV belong to Co–P, the oxidation state of Co, and satellite peaks in the Co 2p_1/2_ spectrum, respectively.^[^ ^22^ ^]^ In the Fe 2p spectrum of Mn‐Co‐Fe‐P, the peaks at 707.13, 710.67, and 714.02 eV belong to Fe–P, the oxidation state of Fe and satellite peaks in Fe 2p_3/2_, respectively.^[^ ^23^ ^]^ Compared with Co 2p and Fe 2p in Co‐Fe‐P, the peaks at 778.93 (Co 2p) and 707.13 eV (Fe 2p) at the low binding energy in Mn‐Co‐Fe‐P are both shifted to the high binding energy.

In the spectrum of Mn 2p, the peaks at 641.71 and 653.81 eV correspond to Mn 2p_3/2_ and Mn 2p_1/2_ in Mn‐Co‐Fe‐P, respectively. The peaks at 641.36 and 653.59 eV belong to Mn^2+^, the peaks at 642.8 and at 654.96 eV belong to Mn^3+^. Compared with Mn‐Co‐Fe‐O, Mn 2p in Mn‐Co‐Fe‐P has an additional satellite peak at 646.38 eV.^[^ ^24^ ^]^ In Figure 3d, the difference of binding energy of Mn 3s in Mn‐Co‐Fe‐O is 4.91 eV, which confirms that the Mn in Mn‐Co‐Fe‐O exists in the form of Mn^3+^ and Mn^4+^, while the Mn in Mn‐Co‐Fe‐P exists in the form of Mn^2+^ and Mn^3+^.^[^ ^25^ ^]^ The characteristic peak at 129.88 eV in the spectrum of P 2p is derived from P in Mn‐Co‐Fe‐P (Figure 3e), and the peak at 133.88 eV is caused by the surface oxidation of the sample exposed to air.^[^ ^26^ ^]^ In the O 1s energy spectrum of Mn‐Co‐Fe‐P, the peak at 531.5 eV indicates the existence of Co–O, Fe–O, Mn–O and P–O, and the peak at 533.02 eV can be expressed as oxidized phosphate species.^[^ ^27^ ^]^

### 2.2 Electrocatalytic Performance of the Heterostructure Mn‐Co‐Fe‐P Nanoarrays

**Figure 4:**
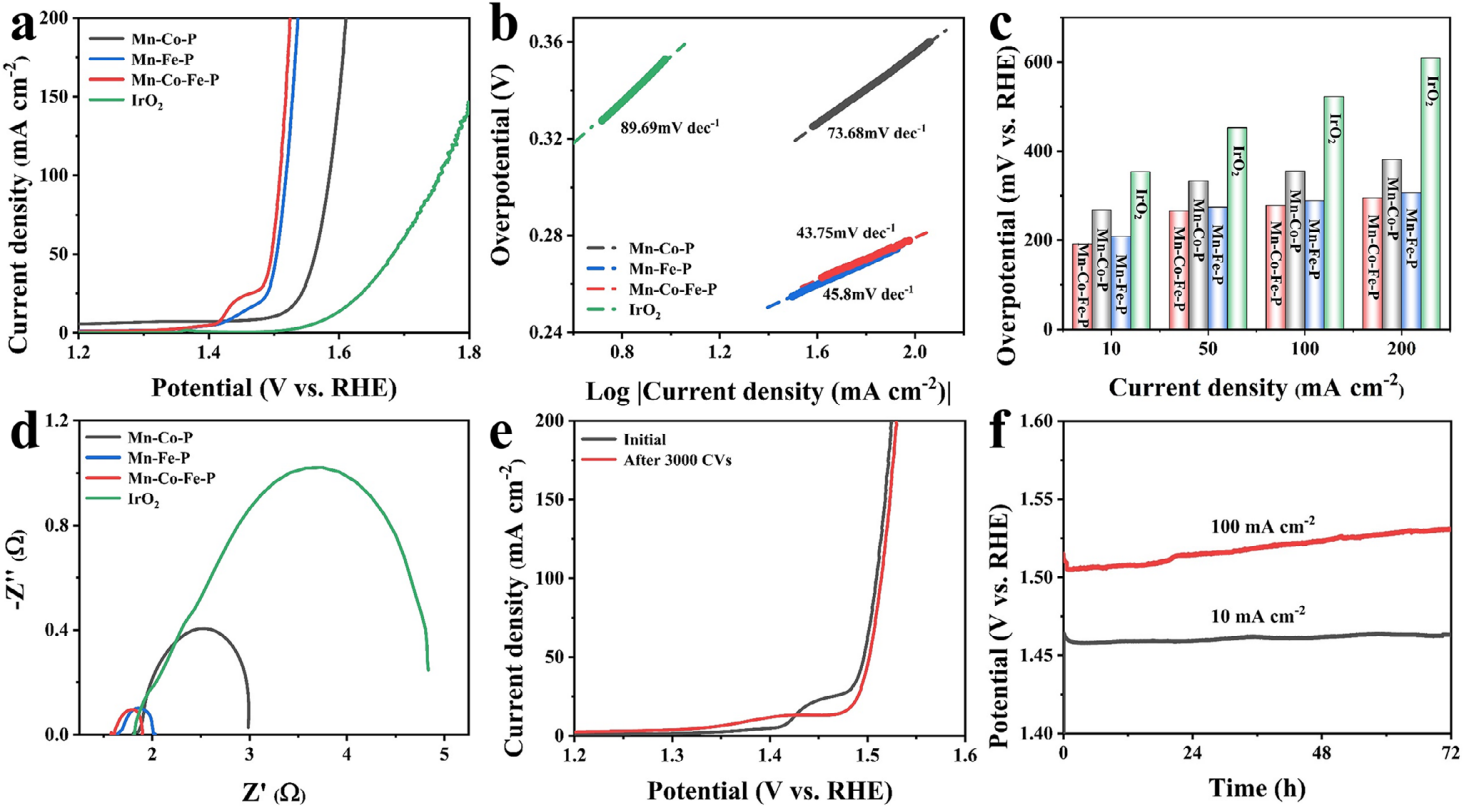
a) LSV curves, b) the corresponding Tafel plots, c) overpotentials at 10, 50, 100, and 200 mA cm^−2^ and d) Nyquist plots of Mn‐Co‐P, Mn‐Fe‐P, Mn‐Co‐Fe‐P nanoarrays and IrO_2_ toward OER; e) LSV curves initially and after 3000 cycles of CV of Mn‐Co‐Fe‐P nanoarrays for OER; f) Chronopotentiometry test of Mn‐Co‐Fe‐P for OER.

The as‐prepared Mn‐Co‐Fe‐P are evaluated using a standard three‐electrode system in 1 m KOH solution to estimate their electrocatalytic performance. A sample slice with a working area 1 × 1 cm^2^ is directly immersed into the solution as the working electrode. The OER performance test results of the heterostructure Mn‐Co‐Fe‐P nanoarrays are shown in **Figure** **4**. According to the LSV curve in Figure 4a–c, the corresponding overpotentials (*η*) of the heterostructure Mn‐Co‐Fe‐P nanoarrays at 10, 50 and 100 mA cm^−2^ current densities are 192, 266, and 279 mV, respectively, which are lower than those of Mn‐Co‐P, Mn‐Fe‐P, and commercial IrO_2_, indicating that the heterostructure Mn‐Co‐Fe‐P nanoarrays have the best OER performance. This is due to the synergistic effect of heterostructure and cobalt‐iron bimetallic, which effectively enhances the electrocatalytic performance. It can be seen that the Tafel slope (Figure 4b) of the heterostructure Mn‐Co‐Fe‐P nanoarrays is the smallest (43.75 mV dec^−1^), which is lower than those of Mn‐Co‐P (73.68 mV dec^−1^), Mn‐Fe‐P (45.8 mV dec^−1^) and IrO_2_ (89.69 mV dec^−1^). As a reference, the OER performance test results of pure nickel foam are presented in previous articles, exhibiting that the catalytic activity of the as‐prepared electrodes all comes from the heterostructure Mn‐Co‐Fe‐P nanoarrays grown on the surface of nickel foam.^[^ ^28^ ^]^ Moreover, the low OER overpotential and the Tafel slope of the heterostructure Mn‐Co‐Fe‐P nanoarrays are better than other electrocatalysts reported previously (Table S2, Supporting Information).

The conductivity of the catalyst can be further evaluated by EIS, and the results are indicated in Figure 4d. The heterostructure Mn‐Co‐Fe‐P nanoarrays has the lowest *Rct*, which manifests that the porous heterostructure Mn‐Co‐Fe‐P can greatly accelerate the electron transfer between the catalyst surface and the reactants for obtaining excellent OER catalytic power.^[^ ^29^, ^30^ ^]^ The OER stability of the catalyst is evaluated by cyclic voltammetry (CV) and chronopotentiometry, as shown in Figure 4e,f. It is seen that the LSV curves of Mn‐Co‐Fe‐P do not change much before and after cycling, demonstrating the high durability of Mn‐Co‐Fe‐P for OER electrocatalysis. Figure 4f reveals that under the conditions of stable current density of 10 and 100 mA cm^−2^, the potential increase is very small within 72 h, testifying that Mn‐Co‐Fe‐P can maintain relatively good stability after long‐term OER catalysis. After the long‐term stability test of the Mn‐Co‐Fe‐P electrocatalyst, it can be observed from Figure S6 (Supporting Information) that the morphology of the Mn‐Co‐Fe‐P electrocatalyst changes after a long period of OER catalysis, which is caused by the transition metal phosphide being oxidized to transition metal phosphate during the OER process. The microstructure and element distribution of Mn‐Co‐Fe‐P after the stability test are characterized by TEM as shown in Figure S7 (Supporting Information). After the OER test, the heterostructure of Mn‐Co‐Fe‐P remains unchanged. However, the elemental distribution shows that the Co, Fe, Mn, and O elements are evenly distributed, while the content of P is reduced to a certain extent, indicating the oxidation process catalyzed by OER. Moreover, previous research reports have demonstrated that metal phosphates play an important role in OER catalysis, and contribute to the stability of the catalytic system during the OER process.^[^ ^31^, ^32^ ^]^

To demonstrate the superior electrocatalytic performance of Mn‐Co‐Fe‐P, the performance of Co–Fe precursor and Mn‐Co‐Fe precursor are compared, as shown in Figure S8 (Supporting Information). Compared with Co–Fe precursor and Mn‐Co‐Fe precursor, Mn‐Co‐Fe‐P has the lowest overpotential and the smallest Tafel slope and *Rct*, indicating that Mn‐Co‐Fe‐P has the ability to form advantages of highly OER active sites. In addition, in order to further verify the admirable electrocatalytic performance of Mn‐Co‐Fe‐P, the OER performance of Mn‐Co‐Fe‐P with different KMnO_4_ concentrations, Co(NO_3_)_2_·6H_2_O and Fe(NO_3_)_3_·9H_2_O ratios, metal precursors and hydrothermal step are also studied. It can be seen from Figures S9 and S10 (Supporting Information) that when the concentration of KMnO_4_ increases or decreases, the catalytic performance of 0.1 Mn‐Co‐Fe‐P and 0.01 Mn‐Co‐Fe‐P does not exceed that of Mn‐Co‐Fe‐P. Meanwhile, the catalytic performance of Mn‐Co‐Fe‐P is also better than that of Mn‐Co_0.75_‐Fe_0.25_‐P and Mn‐Co_0.25_‐Fe_0.75_‐P, testifying that when the concentration of KMnO_4_ is 0.05 m and the molar ratio of Co(NO_3_)_2_·6H_2_O to Fe(NO_3_)_3_·9H_2_O is 1:1, the as‐prepared Mn‐Co‐Fe‐P has the best OER performance. In addition, as shown in Figure S11 (Supporting Information), when Fe(NO_3_)_3_·9H_2_O was replaced by Ni(NO_3_)_2_·6H_2_O, CuCl_2_·2H_2_O, and ZnCl_2_ in the first step of the hydrothermal reaction, respectively, the OER performance of Mn‐Co‐Ni‐P, Mn‐Co‐Cu‐P, and Mn‐Co‐Zn‐P obtained are not ideal, demonstrating that the addition of Fe can improve the catalytic performance. The OER performance of MnCoFe‐P (Figure S12, Supporting Information) synthesized by the one‐step hydrothermal method does not exceed that of Mn‐Co‐Fe‐P, indicating that the 3D structure formed by the two‐step hydrothermal method effectively enhances the catalytic activity.

**Figure 5:**
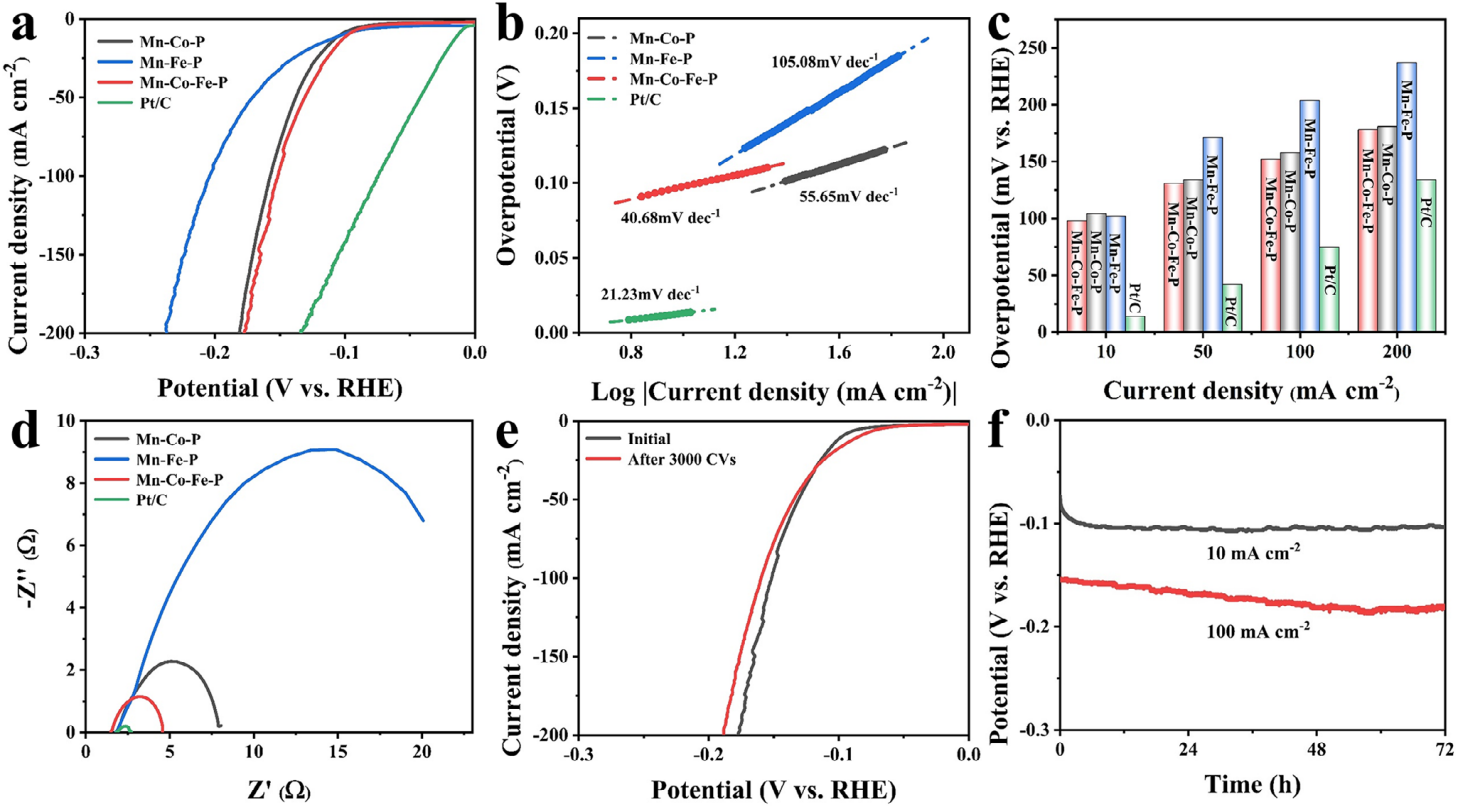
a) LSV curves, b) the corresponding Tafel plots, c) overpotentials at 10, 50, 100, and 200 mA cm^−2^ and d) Nyquist plots of Mn‐Co‐P, Mn‐Fe‐P, Mn‐Co‐Fe‐P nanoarrays and Pt/C toward HER; e) LSV curves initially and after 3000 cycles of CV of Mn‐Co‐Fe‐P nanoarrays for HER; f) Chronopotentiometry test of Mn‐Co‐Fe‐P for HER.

Under the same environment and methods as OER, the HER properties of the catalyst are estimated. As shown in **Figure** **5**, the heterostructure Mn‐Co‐Fe‐P nanoarrays still have admirable HER performance. Typically, when the current density is 10 and 100 mA cm^−2^, the overpotential of Mn‐Co‐Fe‐P is only 98 and 152 mV, respectively, which is lower than that of Mn‐Co‐P and Mn‐Fe‐P. In Figure 5b, the Tafel slope of Mn‐Co‐Fe‐P is 40.68 mV dec^−1^, which is lower than that of Mn‐Co‐P (55.65 mV dec^−1^) and Mn‐Fe‐P (105.08 mV dec^−1^). The smaller Tafel slope proves that there is a good reaction kinetic during the HER process. The measured commercial Pt/C electrode has a lower overpotential with a Tafel slope of 21.23 mV dec^−1^. The test results of pure nickel foam have been given in the previous work^[^ ^28^ ^]^ and the HER performance of the heterostructure Mn‐Co‐Fe‐P nanoarrays are compared with other catalysts reported by previous studies in Table S3 (Supporting Information), indicating that the Mn‐Co‐Fe‐P nanoarrays have admirable HER performance.

The Nyquist plot of Figure 5d reveals that the *Rct* of the heterostructure Mn‐Co‐Fe‐P nanoarrays is smaller than that of other catalysts except Pt/C. It exhibits that Mn‐Co‐Fe‐P has excellent electron transfer ability in the HER electrocatalytic process, which improves the conductivity of the catalyst. Compared with Mn‐Co‐P and Mn‐Fe‐P, Mn‐Co‐Fe‐P has the lowest overpotential, Tafel slope, and *Rct*, testifying that the synergistic effect of Co–Fe bimetallic and heterostructure can improve the electrocatalytic performance. Figure 5e,f reveals the outstanding durability of Mn‐Co‐Fe‐P nanoarrays. The LSV curve obtained after 3000 cycles of the CV cycle test has little change, and the overpotential change of the chronopotentiometry curve is also negligible, explaining that Mn‐Co‐Fe‐P has excellent stability and durability. The FESEM and TEM images of Figures S13 and S14 (Supporting Information) further reveal that the morphology and structure of Mn‐Co‐Fe‐P undergo little change after long‐term durability testing.

Meanwhile, compared with Co–Fe and Mn‐Co‐Fe precursors (Figure S15, Supporting Information), the Mn‐Co‐Fe‐P still exhibits the best HER performance, illustrating that phosphating enables improved HER performance. For comparison, the HER performance of Mn‐Co‐Fe‐P with different KMnO_4_ concentrations, Co(NO_3_)_2_·6H_2_O and Fe(NO_3_)_3_·9H_2_O ratios, metal precursors and hydrothermal step are also investigated, as shown in Figures S16–S19 (Supporting Information). It can be seen from Figures S16 and S17 (Supporting Information) that when the concentration of KMnO_4_ increases or decreases, the catalytic performance of 0.1 Mn‐Co‐Fe‐P and 0.01 Mn‐Co‐Fe‐P catalysts are inferior to Mn‐Co‐Fe‐P. In the meantime, the catalytic performance of Mn‐Co‐Fe‐P is also better than that of Mn‐Co_0.75_‐Fe_0.25_‐P and Mn‐Co_0.25_‐Fe_0.75_‐P, declaring that when the concentration of KMnO_4_ is 0.05 m and the molar ratio of Co(NO_3_)_2_·6H_2_O and Fe(NO_3_)_3_·9H_2_O is 1:1, the as‐prepared Mn‐Co‐Fe‐P has the highest excellent HER performance. In addition, from Figure S18 (Supporting Information), when Fe(NO_3_)_3_·9H_2_O is replaced by Ni(NO_3_)_2_·6H_2_O, CuCl_2_·2H_2_O and ZnCl_2_ in the first step of hydrothermal reaction, the obtained Mn‐Co‐Fe‐P has the best HER performance, which is better than Mn‐Co‐Ni‐P, Mn‐Co‐Cu‐P, and Mn‐Co‐Zn‐P. Furthermore, the HER catalytic performance of 3D heterostructure Mn‐Co‐Fe‐P is better than that of MnCoFe‐P synthesized by one‐step hydrothermal method, as seen in Figure S19 (Supporting Information).

**Figure 6:**
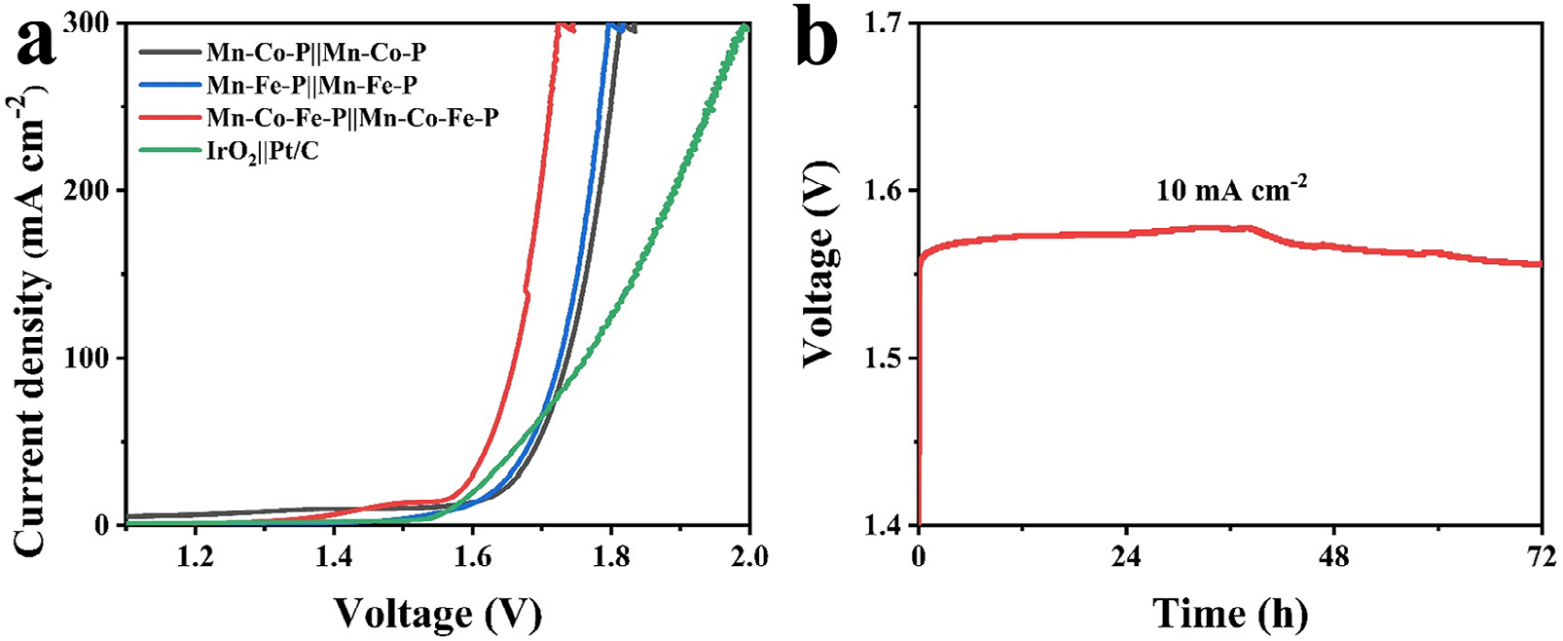
a) Polarization curves of the Mn‐Co‐Fe‐P||Mn‐Co‐Fe‐P cell, Mn‐Co‐P||Mn‐Co‐P cell, Mn‐Fe‐P||Mn‐Fe‐P cell, and the IrO_2_||Pt/C cell for overall water splitting; b) Chronopotentiometry test of Mn‐Co‐Fe‐P||Mn‐Co‐Fe‐P at 10 mA cm^−2^.

Based on the superb OER and HER performance of the heterostructure Mn‐Co‐Fe‐P nanoarrays, the resultant Mn‐Co‐Fe‐P is used as both the cathode and anode of the electrolytic cell, and its overall water splitting performance is measured by using a two‐electrode system. The water splitting LSV curve of the obtained catalyst is shown in **Figure** **6**a. It can be seen that the Mn‐Co‐Fe‐P||Mn‐Co‐Fe‐P full electrolytic cell only needs a voltage of 1.66 V to achieve a current density of 100 mA cm^−2^, which is better than the IrO_2_||Pt/C electrolytic cell. Compared with Mn‐Co‐P and Mn‐Fe‐P, Mn‐Co‐Fe‐P still has the best overall water splitting performance, illustrating that the synergistic effect of cobalt and iron can improve the performance of the catalyst. Furthermore, the heterostructure Mn‐Co‐Fe‐P nanoarrays used for overall water splitting exceed some other reported bifunctional electrocatalysts (Table S4, Supporting Information). The stability of the Mn‐Co‐Fe‐P||Mn‐Co‐Fe‐P full electrolytic cell is tested by chronopotentiometry. Figure 6b exhibits that the voltage fluctuation of Mn‐Co‐Fe‐P nanoarrays after 72 h stability test under 10 mA cm^−2^ current density is very small, demonstrating high stability.

XPS analysis is performed on the Mn‐Co‐Fe‐P nanoarrays after long‐time OER and HER durability tests, as shown in Figure S20 (Supporting Information). Compared with the HER stability test, the strong anodic oxidation in the OER stability test results in an obvious irreversible phase transition of the metal phosphide, accompanied by a change in the morphology of Mn‐Co‐Fe‐P. Obviously, after the OER stability test, the peaks of Co (778.93 eV) and Fe (707.13 eV) at the lower binding energy disappear, which originated from Co‐P and Fe‐P in Mn‐Co‐Fe‐P. This illustrates that the metal phosphide undergoes a phase transition to metal oxides/hydroxides after OER catalysis, which provides active sites for catalytic reactions. Meanwhile, it can be seen from Figure S20c (Supporting Information) that after the OER stability test, the satellite peak of Mn 2p disappears, which means that the state of the multivalent metal Mn has changed. It proves that low‐valent Mn is oxidized to high‐valent Mn. In addition, the disappearance of the peak of metal phosphide in Figure S20d (Supporting Information) attests that the metal phosphide has been oxidized to metal oxide or metal phosphate. This phase transition can also be observed in the O 1s spectrum in Figure S20e (Supporting Information), with a new peak at 529.6 eV corresponding to metal oxides, which is the typical catalytic phase for the reaction. As a result, the new phase formed during the OER process can be considered as the electrocatalytic active phase, which can promote the catalytic process and maintain stable catalytic activity.

For catalysts, large electrochemically active surface area (ECSA) can provide more exposed active sites for the electrocatalytic reaction, thereby improving catalytic performance. ECSA is evaluated by calculating the electrical double layer capacitance (*C*_dl_) from CV measurements at different scan rates. Figure S21a (Supporting Information) is the CV curves of Mn‐Co‐Fe‐P obtained with different scan rates in the potential range of −0.1–0 V versus Ag/AgCl. It can be seen from Figure S21b (Supporting Information) that the *C*_dl_ of the Mn‐Co‐Fe‐P nanoarrays is 10.25 mF cm^−2^ by calculating the slope value of the current density and the scanning speed, and the ECSA is proportional to the *C*_dl_, which can be used to evaluate the ECSA. By rationally designing the 3D heterostructure, a larger specific surface area and active sites can be obtained, and the larger specific surface area is conducive to expanding the contact surface and promoting full contact between the electrode and the electrolyte, thereby improving the catalytic performance.

The catalytic activity of a single active site of the catalyst is calculated by estimating the turnover frequency (TOF) of the catalyst. The CV curve is obtained by cyclic voltammetry in the phosphate buffer solution (PBS) with a concentration of 1.0 m and pH value of 7 to calculate the number of active centers. In Figure S22a,b (Supporting Information), the TOF values of Mn‐Co‐Fe‐P nanoarrays are larger than those of Mn‐Co‐P and Mn‐Fe‐P regardless of OER or HER processes. During the OER process, at 1.53 V, the TOF value of Mn‐Co‐Fe‐P is 4.118 s^−1^, while those of Mn‐Co‐P and Mn‐Fe‐P are only 0.136 and 2.684 s^−1^, respectively, evaluating that the active site of Mn‐Co‐Fe‐P has a high activity of catalyzing OER. In the HER process, the TOF values of Mn‐Co‐Fe‐P, Mn‐Co‐P, and Mn‐Fe‐P are 0.092, 0.078, and 0.047 s^−1^ at −0.15 V, respectively. Mn‐Co‐Fe‐P has the highest TOF value, explaining that its active site has the uppermost catalytic activity for HER.

### 2.3 DFT Calculation of the Heterostructure Mn‐Co‐Fe‐P Nanoarrays

**Figure 7:**
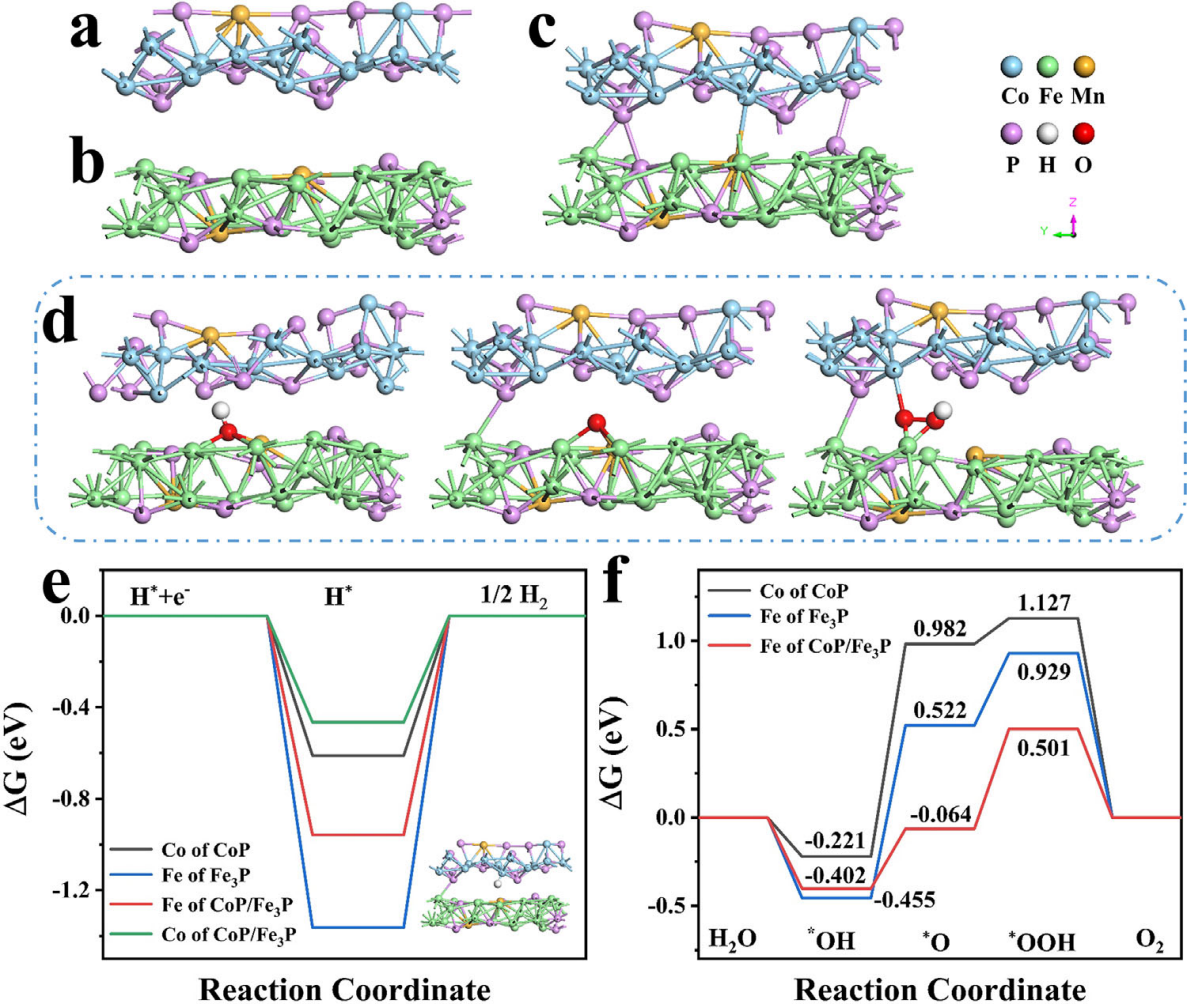
Slab model of a) CoP (111), b) Fe_3_P (321), and c) CoP/Fe_3_P, d) diagrams show the OER pathway, Δ*G* of e) HER, and f) OER.

To further prove the wonderful catalytic activity and stability of the heterostructure Mn‐Co‐Fe‐P nanoarrays, the density functional theory (DFT) calculations of Mn‐Co‐Fe‐P are carried out, as shown in **Figure** **7**. The (111) crystal plane of CoP and the (321) crystal plane of Fe_3_P are selected to calculate the Gibbs free energy, and the Gibbs free energy of the heterostructure CoP/Fe_3_P is also calculated. It is seen from Figure 7a–c that there exist the (111) crystal plane of CoP, the (321) crystal plane of Fe_3_P, and the heterostructure CoP/Fe_3_P, respectively. Figure 7e shows the adsorption‐free energy step diagram during the HER process of CoP, Fe_3_P, and CoP/Fe_3_P. It can be seen that the Δ*G* of the Co‐active site in the heterostructure CoP/Fe_3_P is −0.465 eV, the Δ*G* of the Fe active site in the heterostructure CoP/Fe_3_P is −0.958 eV, the Δ*G* of the Co‐active site in the CoP is −0.611 eV, and the *ΔG* of the Fe active site in Fe_3_P is −1.363 eV. Among them, the Δ*G* of the Co‐active site in the heterostructure CoP/Fe_3_P is the smallest, and the heterostructure Mn‐Co‐Fe‐P has enhanced catalytic activity, which further illustrates that the construction of the heterostructure can improve the catalytic performance.

The OER process is more complex than the HER process, and the four steps are the adsorption free energy barriers of the four sub‐reaction steps in the OER reaction. Figure 7d illustrates the Mn‐Co‐Fe‐P adsorbing ^*^OH, ^*^O, and ^*^OOH intermediates during the OER process. Figure 7f is the OER step diagram of the Fe active site in the heterostructure CoP/Fe_3_P, the Fe active site in Fe_3_P and the Co active site in CoP under alkaline conditions. It can be seen that the reaction barriers in the three models all come from the intermediate two steps, because the reaction free energy barriers of the first step and the fourth step are negative, proving that these two processes are spontaneous. In the second step (OH^*^→O^*^), the reaction free energy barrier of the Fe active site in the heterostructure CoP/Fe_3_P is 0.338 eV, while the reaction free energy barriers of the Fe active site in Fe_3_P and the Co active site in CoP are 1.007 and 1.203 eV, respectively. In the third step (O^*^→OOH^*^), the reaction free energy barrier of the Fe active site in the heterostructure CoP/Fe_3_P is 0.565 eV, and the reaction free energy barriers of the Fe active site in Fe_3_P and the Co active site in CoP are 0.407 and 0.145 eV, respectively. For the heterostructure CoP/Fe_3_P, the third step is the rate‐determining step of its OER, while in CoP and Fe_3_P, the second step is the rate‐determining step of their OER. The reaction free energy barrier of the rate‐determining step of the heterostructure CoP/Fe_3_P is the lowest. In addition, the reaction free energy barriers of the second and third steps of the heterostructure CoP/Fe_3_P are the smallest, exhibiting that the heterostructure significantly improves the catalytic activity of OER and can drive the OER reaction with the lowest overpotential.

## 3 Conclusion

In summary, the heterostructure Mn‐Co‐Fe‐P nanoarrays were successfully fabricated on self‐supporting nickel foam by a facile hydrothermal method and phosphating method. In situ growth improves the stability and conductivity of Mn‐Co‐Fe‐P electrocatalysts, and the 3D “sheet–sheet” structure expands the specific surface area of the catalyst and exposes more active sites, endowing Mn‐Co‐Fe‐P outstanding electrocatalytic properties. Theoretical calculations certify that in OER, the Fe active site in heterostructure CoP/Fe_3_P promotes the catalytic process, while in HER, the Co active site in heterostructure CoP/Fe_3_P promotes the catalytic process, demonstrating that the synergistic effect of the Fe and Co makes the catalyst have the admirable bifunctional electrocatalytic performance. This study provides a practical route to develop bifunctional electrocatalysts for overall water splitting.

## 4 Experimental Section

### Materials and Preparation Procedure

All chemicals were analytical grade and used as received. Before all the synthesis processes, one piece of nickel foam (NF, 3 × 2 cm^2^) was cleaned with 3 m hydrochloric acid, absolute ethanol, and deionized water successively for several minutes, respectively.

### Materials and Preparation Procedure—Synthesis of Co–Fe Precursor

Co(NO_3_)_2_·6H_2_O (0.5 mmol), 0.5 mmol Fe(NO_3_)_3_·9H_2_O, 2 mmol NH_4_F, and 4 mmol urea were dissolved in 25 mL water under vigorous stirring to afford a homogeneous solution. Subsequently, the solution was transferred into a Teflon‐lined stainless steel autoclave with one piece of nickel foam immersed into the solution and sealed and maintained at 120 °C for 3 h. After the reaction, the autoclave was cooled down to room temperature, and the nickel foam was washed with deionized water and dried at 70 °C in a vacuum oven to obtain the Co–Fe precursor on nickel foam support.

### Materials and Preparation Procedure—Synthesis of Mn‐Co‐Fe Precursor

Mn‐Co‐Fe precursor was prepared via a facile hydrothermal method. Typically, 0.05 m KMnO_4_ solution was placed in a Teflon‐lined stainless steel autoclave with one piece of Co‐Fe precursor obtained by the previous step. After reacting at 120 °C for 1 h, the resultant Mn‐Co‐Fe precursor on nickel foam was washed with deionized water several times, and dried in a vacuum oven at 70 °C overnight.

### Materials and Preparation Procedure—Synthesis of Mn‐Co‐Fe‐P

First of all, a porcelain crucible boat loaded with Mn‐Co‐Fe precursor was placed on the downstream side of the furnace, and another boat loaded with 300 mg NaH_2_PO_2_ was placed on the upstream side of the furnace. Subsequently, the Mn‐Co‐Fe precursor was heated to 350 °C for 2 h with a heating rate of 2 °C min^−1^ under an Ar atmosphere. The as‐prepared Mn‐Co‐Fe‐P was obtained after cooling to ambient temperature.

As a comparison, in the preparation process, the amount of cobalt and iron was changed from 0.5 and 0.5 mmol to 0.75 and 0.25 mmol, and 0.25 and 0.75 mmol, respectively, and the as‐obtained samples were named Mn‐Co_0.75_‐Fe_0.25_‐P and Mn‐Co_0.25_‐Fe_0.75_‐P. The concentration of KMnO_4_ solution was changed to 0, 0.01, and 0.1 m, respectively, and the samples were named Co‐Fe‐P, 0.01 Mn‐Co‐Fe‐P and 0.1 Mn‐Co‐Fe‐P. Fe(NO_3_)_3_·9H_2_O in the first hydrothermal reaction was changed to Ni(NO_3_)_2_·6H_2_O, ZnCl_2_ and CuCl_2_·2H_2_O, and the samples obtained were named Mn‐Co‐Ni‐P, Mn‐Co‐Zn‐P and Mn‐Co‐Cu‐P, respectively. In addition, Mn‐Co‐P and Mn‐Fe‐P were obtained without adding Fe(NO_3_)_3_·9H_2_O and Co(NO_3_)_2_·6H_2_O in the first step of the hydrothermal process, respectively, and other preparation conditions remained unchanged. Finally, 0.05 m KMnO_4_ was added in the first step of the hydrothermal process, and the second step of the hydrothermal process was not carried out. The other preparation conditions were unchanged and the MnCoFe‐P was obtained by one‐step hydrothermal method.

### Characterization of Electrocatalysts

The X‐ray diffraction (XRD) analysis was obtained by powder X‐ray diffraction using Cu Kα radiation (Empyrean 200 895, PANalytical B.V.). Field emission scanning electron microscopy (FESEM) and transmission electron microscopy (TEM) investigations were carried out on a Hitachi SU8010 and a Hitachi 7700 equipped with an energy dispersive X‐ray (EDX) spectrometer. High‐resolution transmission electron microscopy (HRTEM) was performed on a TEM (Tecnai G2 F20). X‐ray photoelectron spectroscopy (XPS) was processed on an ESCALAB 250XI. The contents of Co, Fe, Mn, and P were evaluated using inductively coupled plasma‐mass spectrometry (ICP‐MS).

### Electrochemical Measurements of Electrocatalysts

All electrochemical tests were performed at ambient conditions. The experiments for the related HER and OER reactions were performed using an electrochemical workstation (Model CHI660E, Shanghai Chenhua Instrument Co., Ltd.) with a standard three‐electrode system in 1 m KOH (pH 14) at room temperature. The Ag/AgCl was used as the reference electrode, and a Pt sheet and the as‐prepared catalyst (1 × 1 cm^2^) were used as the counter and the working electrodes, respectively. The reference electrode was calibrated before use.^[^ ^33^ ^]^ All potentials were converted to the reversible hydrogen electrode (RHE) potential using the equation: *E_RHE_* = *E*_*Ag*/*AgCl*_  + 0.059 × *pH* + 0.197. Linear sweep voltammetry (LSV) was performed at a scan rate of 1 mV s^−1^. Electrochemical impedance spectroscopy (EIS) was performed at 0.56 V versus Ag/AgCl (−1.10 V) for the OER (HER). The double layer capacitance (*C*_dl_) and electrochemical surface area (ECSA) were obtained using cyclic voltammetry (CV) at scan rates of 20–100 mV s^−1^ in the range of −0.10–0 V versus Ag/AgCl. All polarisation curves were corrected using *iR* compensation. Turnover frequency (*TOF*) could be calculated with the previously reported equation^[^ ^34^ ^]^: *TOF*  =  *j*/*Q*. Where *Q* is the integral charges, and *j* is the current density. In this work, *TOF* is gathered from the CV measurements conducted at a scan rate of 50 mV s^−1^ with the potential range of 0.9–1.7 V (−0.2–0.6 V) in 1.0 m phosphate buffer solution (PBS, pH 7) for OER (HER).

### DFT Calculation of Electrocatalysts

All the calculations were performed in the framework of the density functional theory with the projector augmented plane‐wave method, as implemented in the Vienna Ab‐initio Simulation Package (VASP). The generalized gradient approximation proposed by Perdew, Burke, and Ernzerhof was selected for the exchange‐correlation potential. The Van der Waals interaction was described by the DFT‐D3 approach. The cut‐off energy for plane wave was set to 400 eV. A vacuum layer of 15 Å was added perpendicular to the sheet to avoid artificial interaction between periodic images. The Brillouin zone integration was performed using a 2 × 2 × 1 K‐mesh. All the structures were relaxed until the residual forces on the atoms have declined to less than 10^−4^ eV Å^−1^.

## Conflict of Interest

The authors declare no conflict of interest.

## Supporting information



Supporting Information

## Data Availability

The data that support the findings of this study are available from the corresponding author upon reasonable request.
