# Supplementary material for: Constructing Mn‐Co‐Fe Ternary Metal Phosphides Nanosheet Arrays as Bifunctional Electrocatalysts for Overall Water Splitting
Source: Adv Sci (Weinh). 2025 Mar 27;12(22):2417521. doi: 10.1002/advs.202417521 (PMC12165077; doi:10.1002/advs.202417521)
Supplement: Supplementary file 1 — Supporting Information [file ADVS-12-2417521-s001.docx]

Supporting Information

**Constructing Mn-Co-Fe Ternary Metal Phosphides Nanosheet Arrays as Bifunctional** **Electrocatalysts for Overall Water Splitting**

*Fan Wang^a, b^, Zhen Pei^a^, Zhou Xu^a^, Tengteng Qin^a^, Xin Ouyang^a^, Dongyun Li^b^, Yang Hou^c^ and Xingzhong Guo^a, d^**

*^a^* *State Key Laboratory of Silicon and Advanced Semiconductor Materials, School of Materials Science and Engineering, Zhejiang University, Hangzhou 310058, China*

*^b^ College of Materials and Chemistry, China Jiliang University, Hangzhou 310018, China*

*^c^ Key Laboratory of Biomass Chemical Engineering of Ministry of Education College of Chemical and Biological Engineering, Zhejiang University, Hangzhou 310027, China*

*^d^ Hangzhou Global Scientific and Technological Innovation Center, Zhejiang University, Hangzhou 311200, China*

*Corresponding E-mail: msewj01@zju.edu.cn;*


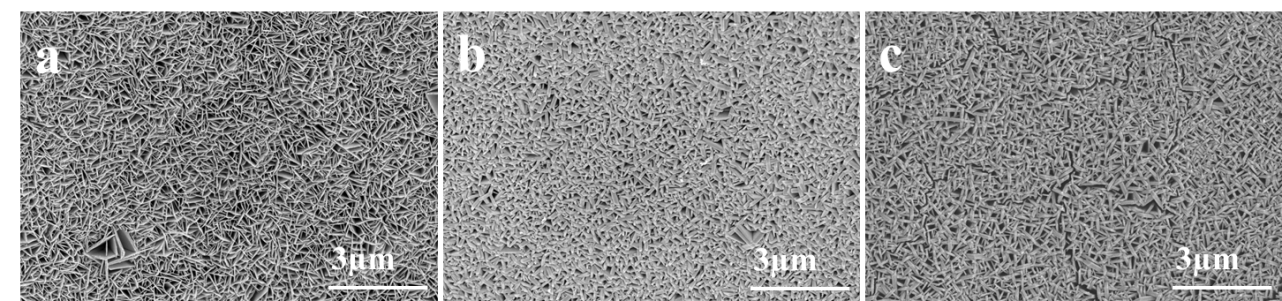
Figure S1 FESEM images of (a) Co-Fe precursor, (b) Mn-Co-Fe precursor and (c) Mn-Co-Fe-P.


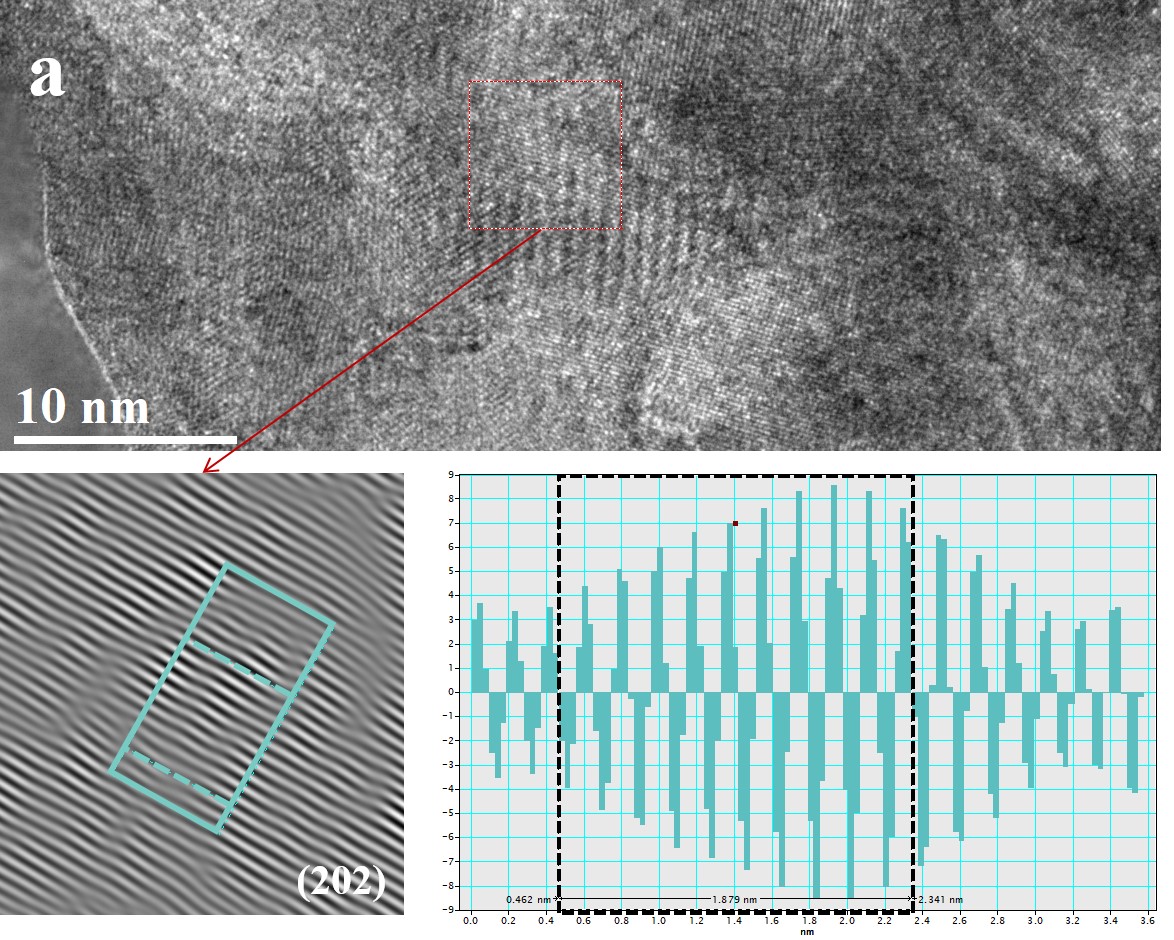


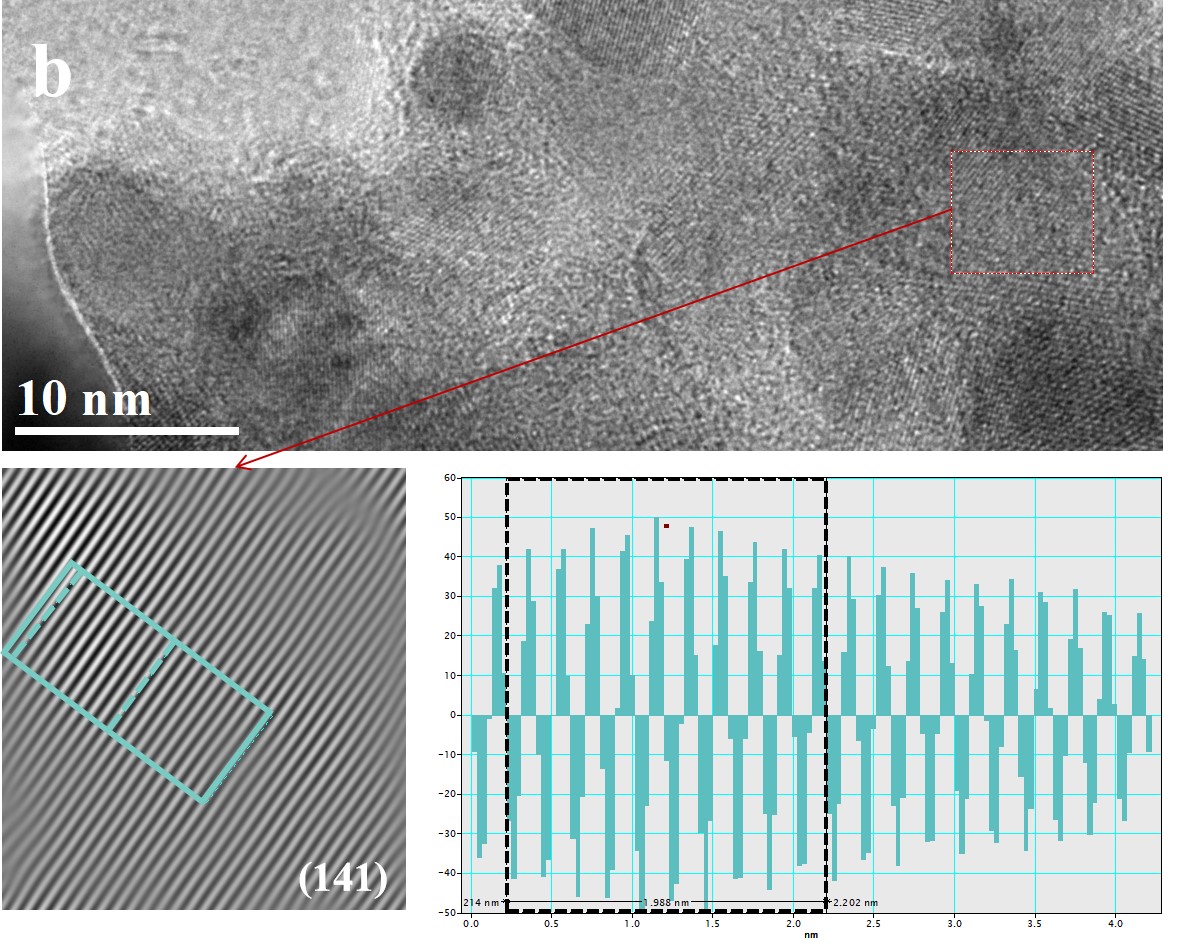


Figure S2 HRTEM images of Mn-Co-Fe-P.


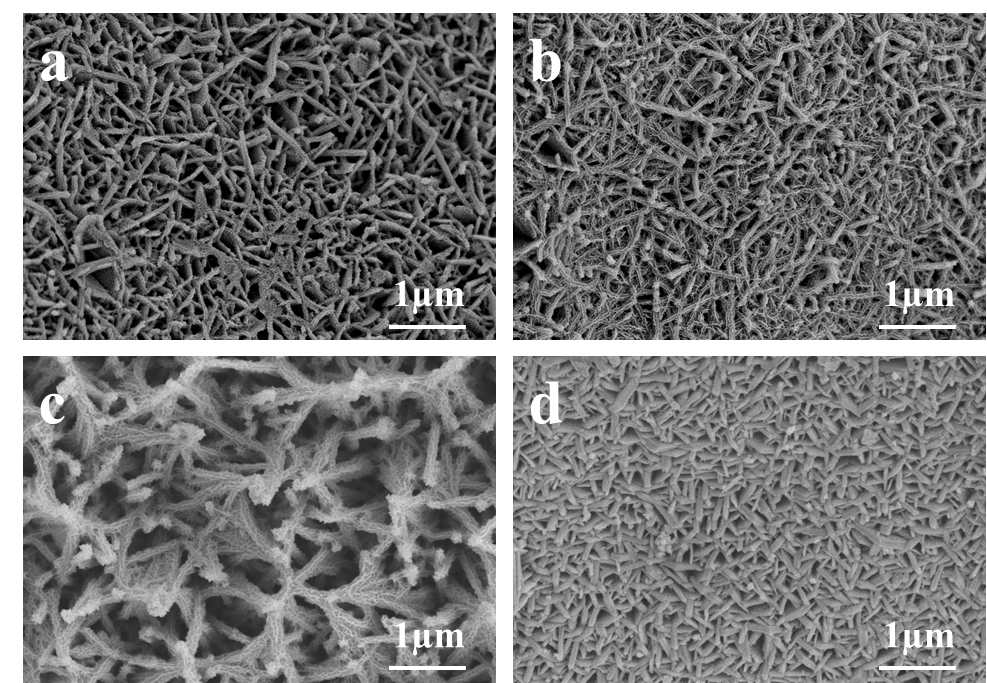


Figure S3 FESEM images of (a) 0.01 Mn-Co-Fe-P, (b) 0.1 Mn-Co-Fe-P, (c) Mn-Co_0.75_-Fe_0.25_-P and (d) Mn-Co_0.25_-Fe_0.75_-P.


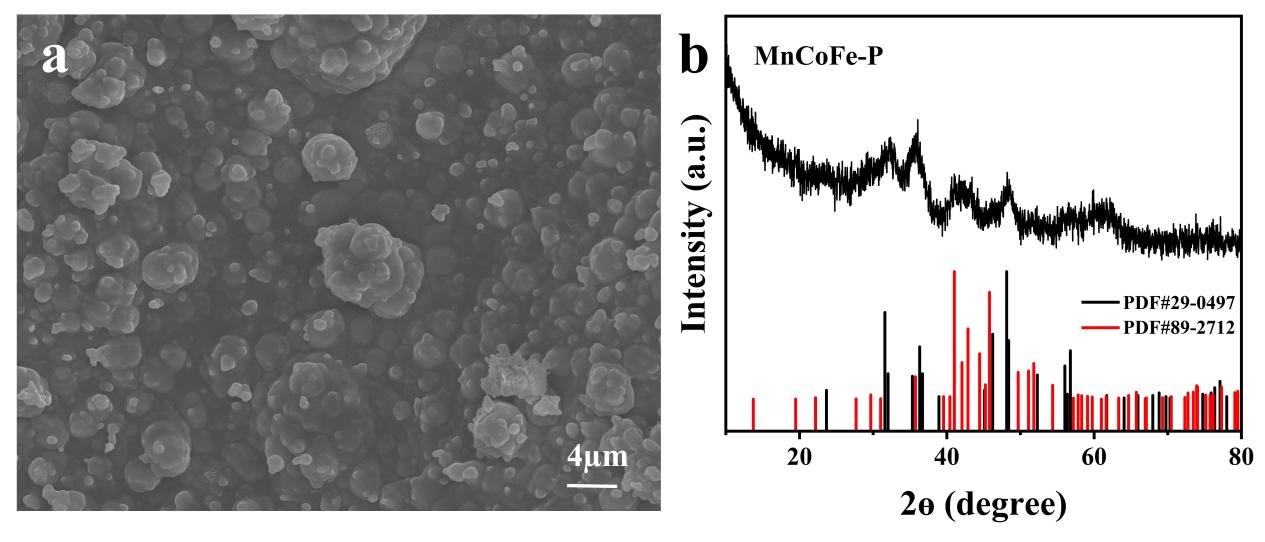


Figure S4 (a) FESEM image and (b) XRD pattern of MnCoFe-P.

Table S1 Metal-atomic content detected by ICP-MS analysis.

| Catalysts | Mn (at. %) | Co (at. %) | Fe (at. %) | | P (at. %) |
| --- | --- | --- | --- | --- | --- |
| Mn-Co-Fe-P | 0.93 | 1.21 | 1.23 | 3 | |


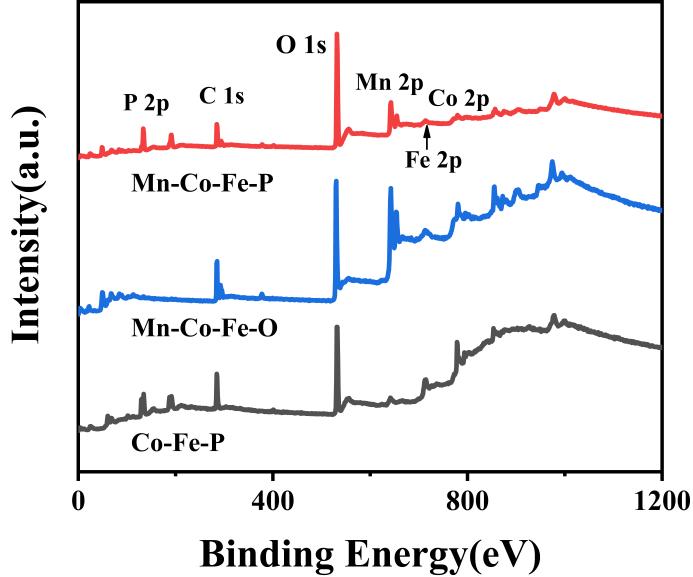


Figure S5 Overall XPS spectra of Co-Fe-P, Mn-Co-Fe-O and Mn-Co-Fe-P.

Table S2 Comparison of the OER performances of the heterostructure Mn-Co-Fe-P nanoarrays with the previously reported electrocatalysts at alkaline media.

| Electrocatalysts | Overpotential (mV)  at 10 mA cm^-2^ | Overpotential (mV)  at 100 mA cm^-2^ | Tafel slop  (mV dec^-1^) | Reference |
| --- | --- | --- | --- | --- |
| heterostructure Mn-Co-Fe-P nanoarrays | 192 | 279 | 43.75 | This work |
| Fe-CoP/CoO | 219 |  | 52 | [1] |
| HOF-Co_0.5_Fe_0.5_/NF | 278 |  | 59 | [2] |
| (CoFe)(OH)_x_ | 275 |  | 34 | [3] |
| CoFe-LDH/NF | 250 |  | 35 | [4] |
| CoFeBiP | 273 |  | 77.3 | [5] |
| Co-Fe oxyphosphide | 280 |  | 53 | [6] |
| CoMn-LDHs | 395 |  | 45 | [7] |
| MnO/Co/PGC | 301 |  | 77 | [8] |
| MnCoP/CC | 261 | 460 | 44.9 | [9] |
| Mn_0.6_Co_0.4_P-rGO | 250 |  | 65 | [10] |
| Ni-Fe-K_0.23_MnO_2_ CNFs-300 | 270 | 320 | 42.3 | [11] |
| N-CoO@CoP |  | 332 | 81.5 | [12] |


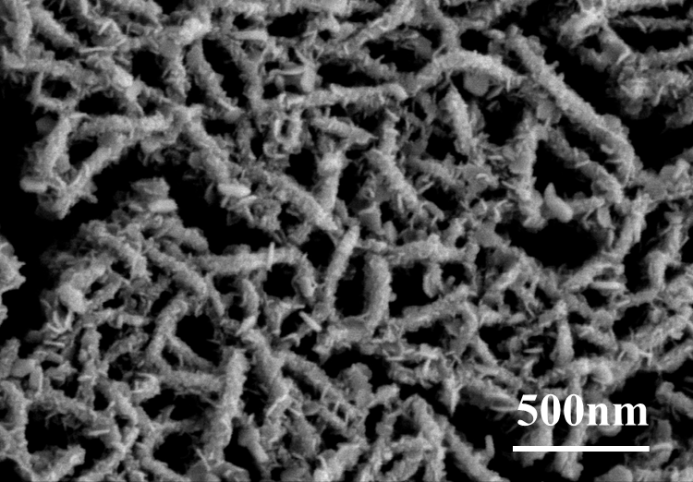


Figure S6 FESEM images of Mn-Co-Fe-P after 72 h chronopotentiometry test towards OER.


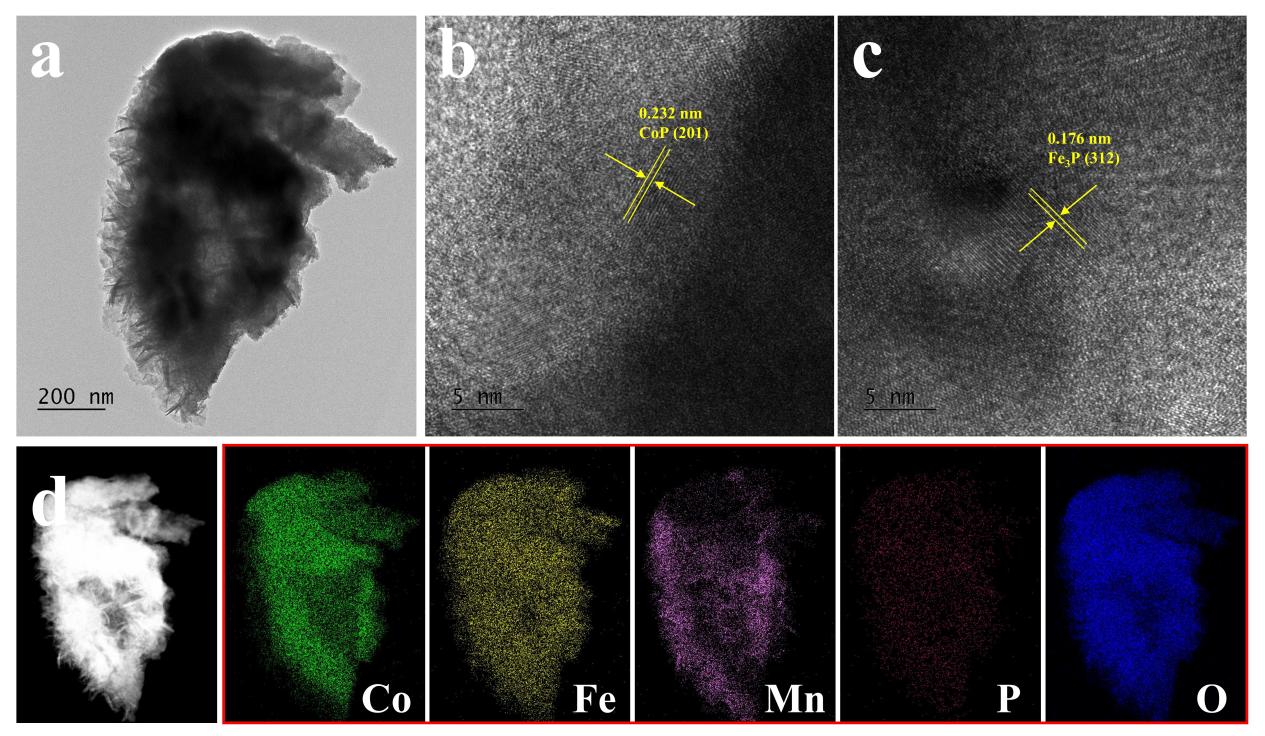


Figure S7 (a) TEM, (b-c) HRTEM and (d) elemental mapping images of Mn-Co-Fe-P after 72 h chronopotentiometry test towards OER.


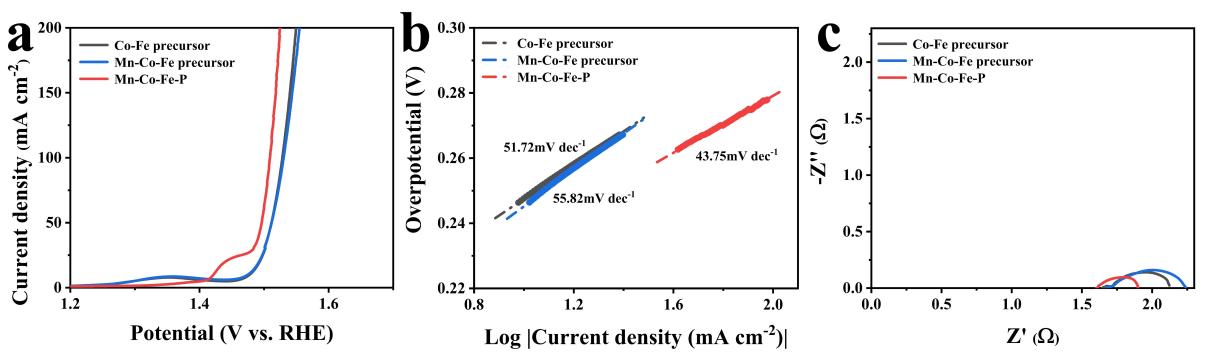


Figure S8 (a) LSV curves, (b) the corresponding Tafel plots and (c) Nyquist plots of Co-Fe precursor, Mn-Co-Fe precursor and Mn-Co-Fe-P nanoarrays towards OER.


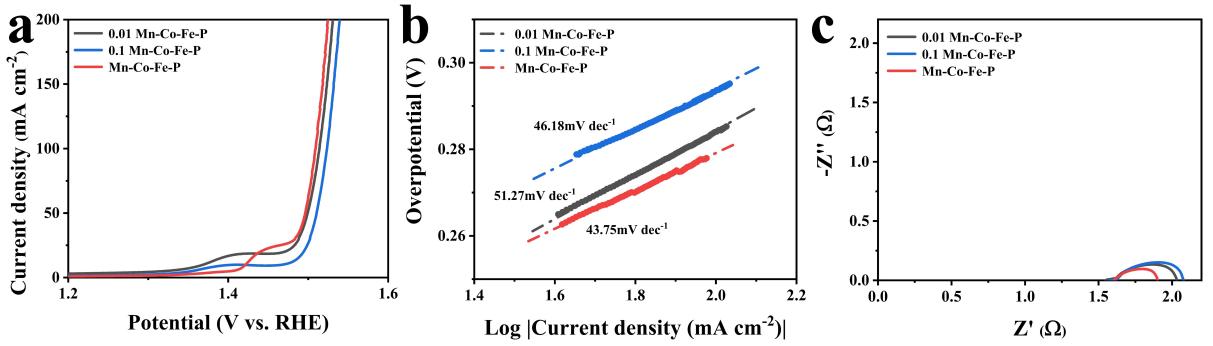


Figure S9 (a) LSV curves, (b) the corresponding Tafel plots and (c) Nyquist plots of 0.01 Mn-Co-Fe-P, 0.1 Mn-Co-Fe-P and Mn-Co-Fe-P nanoarrays towards OER.


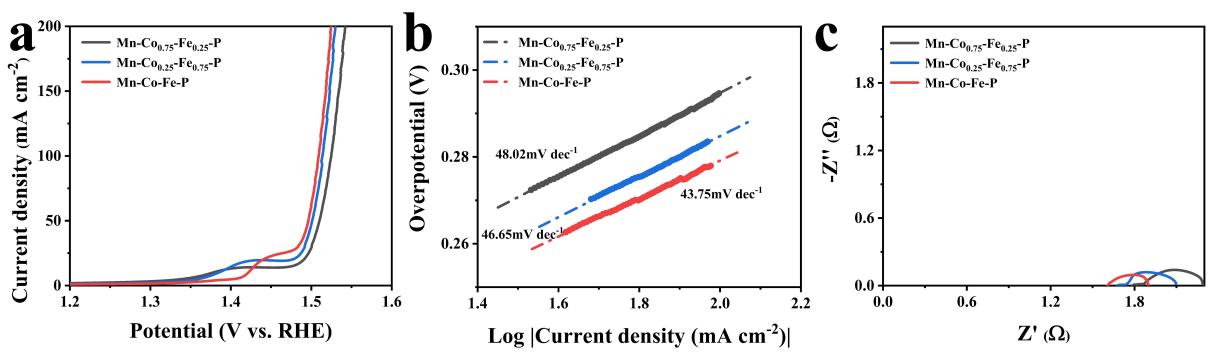


Figure S10 (a) LSV curves, (b) the corresponding Tafel plots and (c) Nyquist plots of Mn-Co_0.75_-Fe_0.25_-P, Mn-Co_0.25_-Fe_0.75_-P and Mn-Co-Fe-P nanoarrays towards OER.


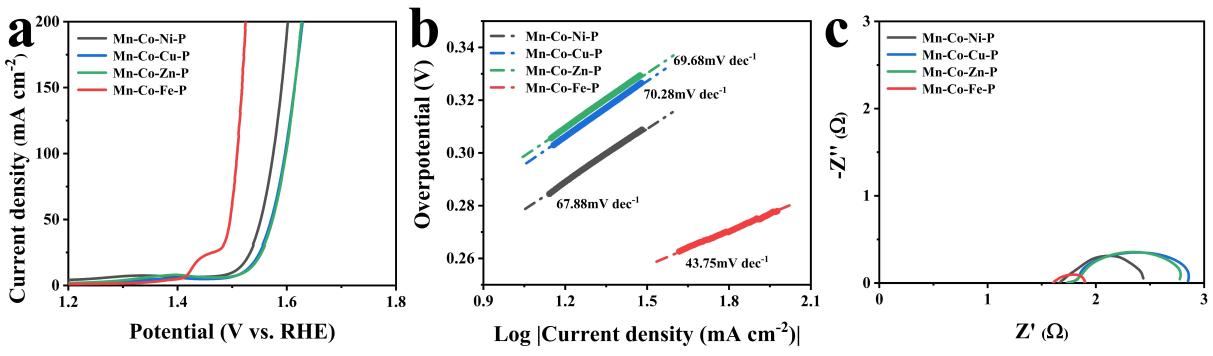


Figure S11 (a) LSV curves, (b) the corresponding Tafel plots and (c) Nyquist plots of Mn-Co-Ni-P, Mn-Co-Cu-P, Mn-Co-Zn-P and Mn-Co-Fe-P nanoarrays towards OER.


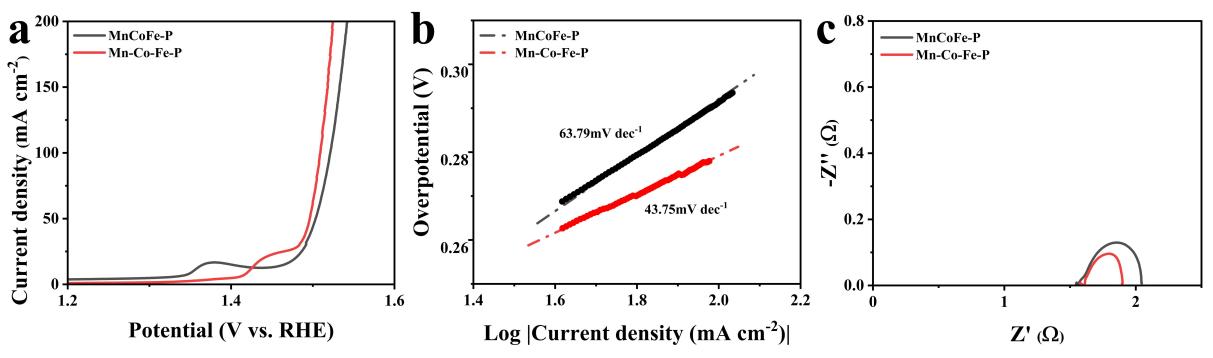


Figure S12 (a) LSV curves, (b) the corresponding Tafel plots and (c) Nyquist plots of MnCoFe-P and Mn-Co-Fe-P nanoarrays towards OER.

Table S3 Comparison of the HER performances of the heterostructure Mn-Co-Fe-P nanoarrays with the previously reported electrocatalysts at alkaline media.

| Electrocatalysts | Overpotential (mV)  at 10 mA cm^-2^ | Overpotential (mV)  at 100 mA cm^-2^ | Tafel slop  (mV dec^-1^) | Reference |
| --- | --- | --- | --- | --- |
| heterostructure Mn-Co-Fe-P nanoarrays | 98 | 152 | 40.68 | This work |
| HOF-Co_0.5_Fe_0.5_/NF | 170 |  | 137 | [2] |
| Co-Fe oxyphosphide | 180 |  | 62 | [6] |
| Ni-Fe-K_0.23_MnO_2_ CNFs-300 | 116 | 242 | 103.9 | [11] |
| N-CoO@CoP |  | 201 | 37 | [12] |
| Mn-N-Co_9_S_8_ | 102 | 238 | 107.2 | [13] |
| Mn-CoP nanosheets | 195 |  | 69 | [14] |
| Mn doped CoP | 100 |  | 53 | [15] |
| CoNiMn/NC | 191 |  | 64.38 | [16] |
| CoMn-P@NG | 164 |  | 111 | [17] |
| Mn-O@CoP | 106 |  | 56 | [18] |


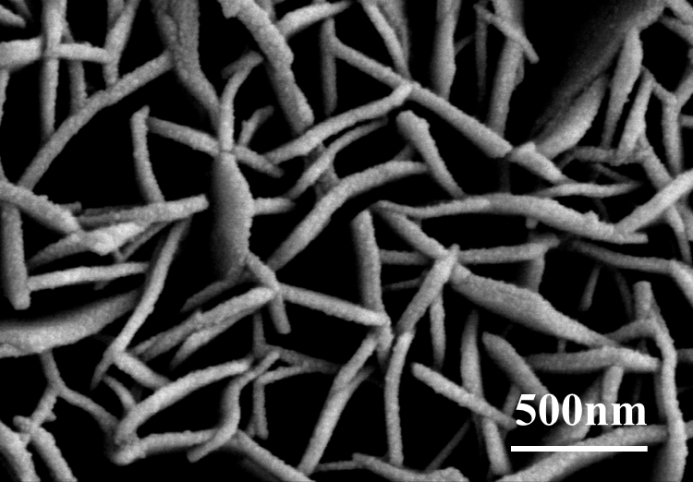


Figure S13 FESEM images of Mn-Co-Fe-P after 72 h chronopotentiometry test towards HER.


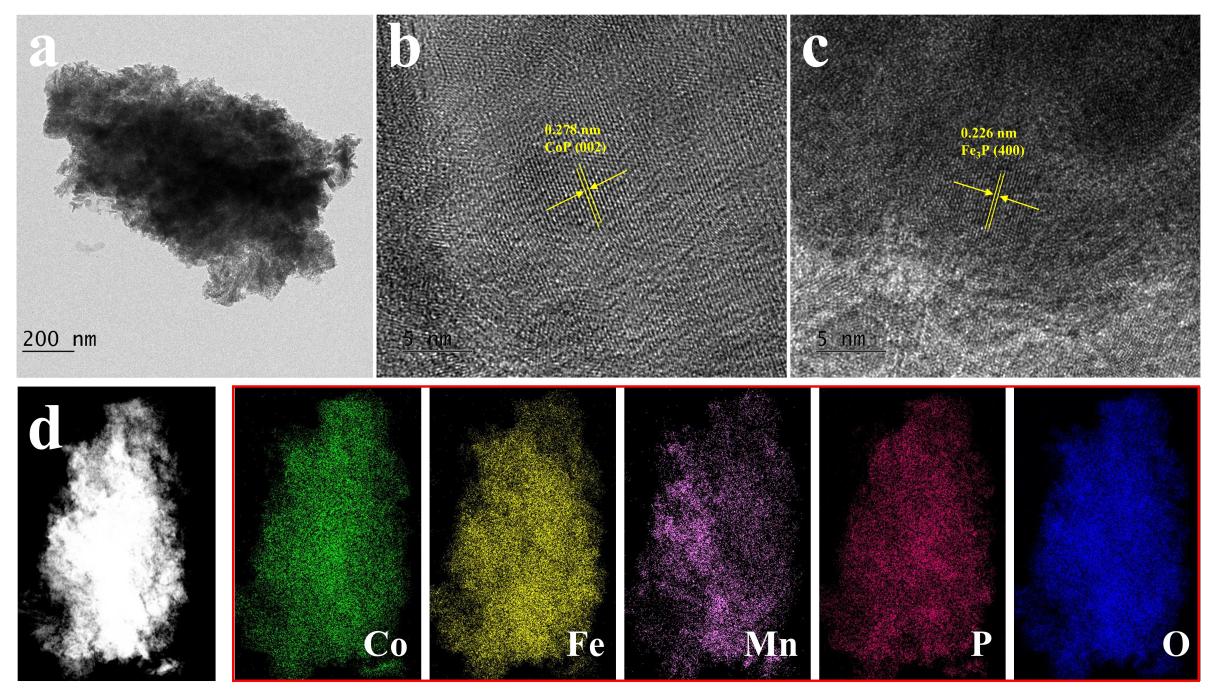


Figure S14 (a) TEM, (b-c) HRTEM and (d) elemental mapping images of Mn-Co-Fe-P after 72 h chronopotentiometry test towards HER.


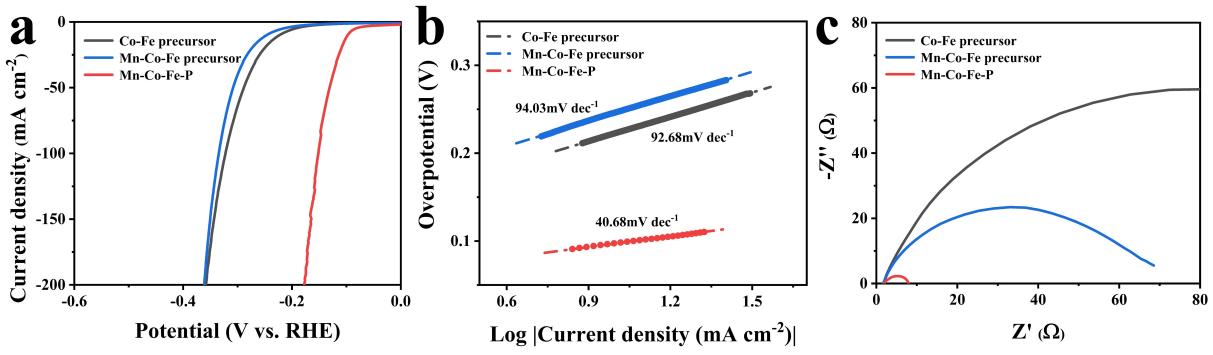


Figure S15 (a) LSV curves, (b) the corresponding Tafel plots and (c) Nyquist plots of Co-Fe precursor, Mn-Co-Fe precursor and Mn-Co-Fe-P nanoarrays towards HER.


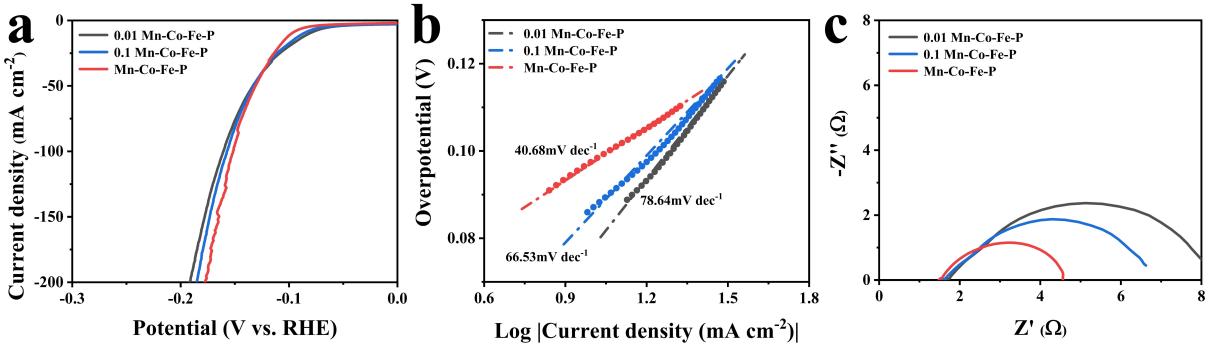


Figure S16 (a) LSV curves, (b) the corresponding Tafel plots and (c) Nyquist plots of 0.01 Mn-Co-Fe-P, 0.1 Mn-Co-Fe-P and Mn-Co-Fe-P nanoarrays towards HER.


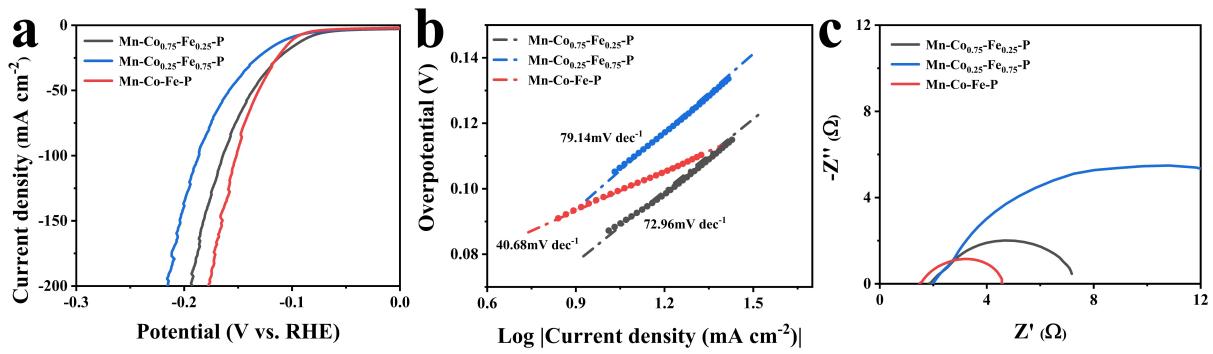


Figure S17 (a) LSV curves, (b) the corresponding Tafel plots and (c) Nyquist plots of Mn-Co_0.75_-Fe_0.25_-P, Mn-Co_0.25_-Fe_0.75_-P and Mn-Co-Fe-P nanoarrays towards HER.


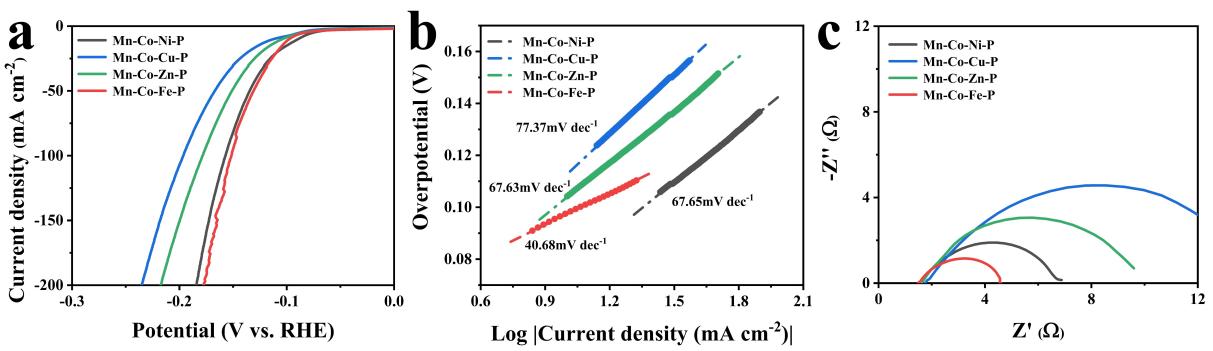


Figure S18 (a) LSV curves, (b) the corresponding Tafel plots and (c) Nyquist plots of Mn-Co-Ni-P, Mn-Co-Cu-P, Mn-Co-Zn-P and Mn-Co-Fe-P nanoarrays towards HER.


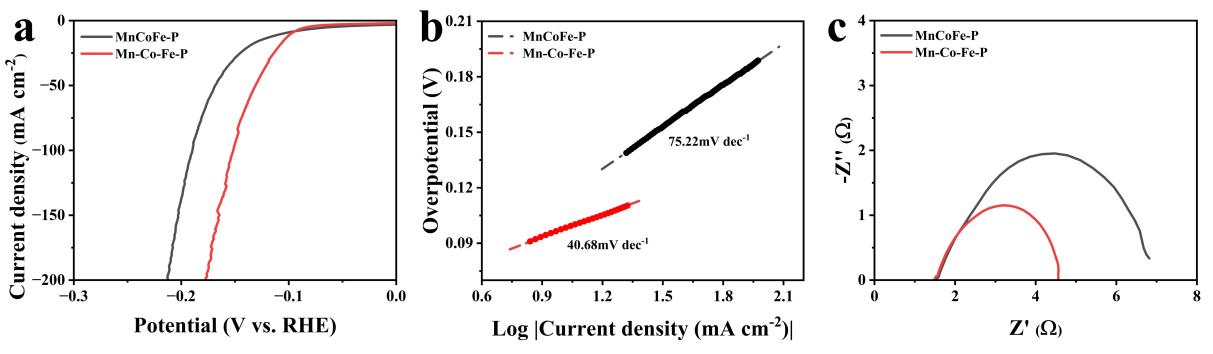


Figure S19 (a) LSV curves, (b) the corresponding Tafel plots and (c) Nyquist plots of MnCoFe-P and Mn-Co-Fe-P nanoarrays towards HER.

Table S4 Comparison of overall water splitting performances of the heterostructure Mn-Co-Fe-P nanoarrays with previously reported electrocatalysts at alkaline media.

| Electrocatalysts | Voltage (V)  at 10 mA cm^-2^ | Voltage (V)  at 100 mA cm^-2^ | Reference |
| --- | --- | --- | --- |
| heterostructure Mn-Co-Fe-P nanoarrays |  | 1.66 | This work |
| HOF-Co_0.5_Fe_0.5_/NF | 1.63 |  | [2] |
| CoFe-P//CoFe-LDH | 1.51 |  | [4] |
| Co-Fe oxyphosphide | 1.69 |  | [6] |
| Ni-Fe-K_0.23_MnO_2_ CNFs-300 | 1.62 | 1.81 | [11] |
| N-CoO@CoP |  | 1.79 | [12] |
| CoMn-LDH@g-C_3_N_4_ | 1.62 |  | [19] |
| Cu@CoP | 1.65 |  | [20] |
| Mn-Co-P | 1.74 |  | [21] |


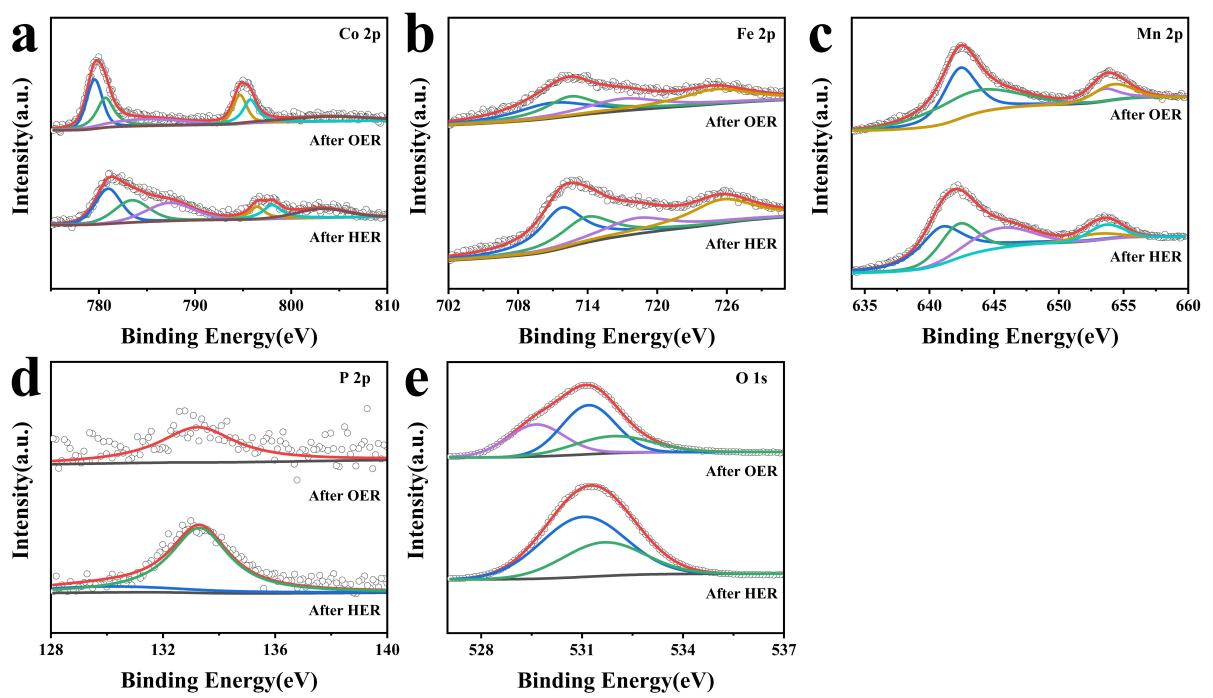


Figure S20 XPS spectra of (a) Co 2p, (b) Fe 2p, (c) Mn 2p, (d) P 2p and (e) O 1s of Mn-Co-Fe-P nanoarrays after HER stability test and after OER stability test.


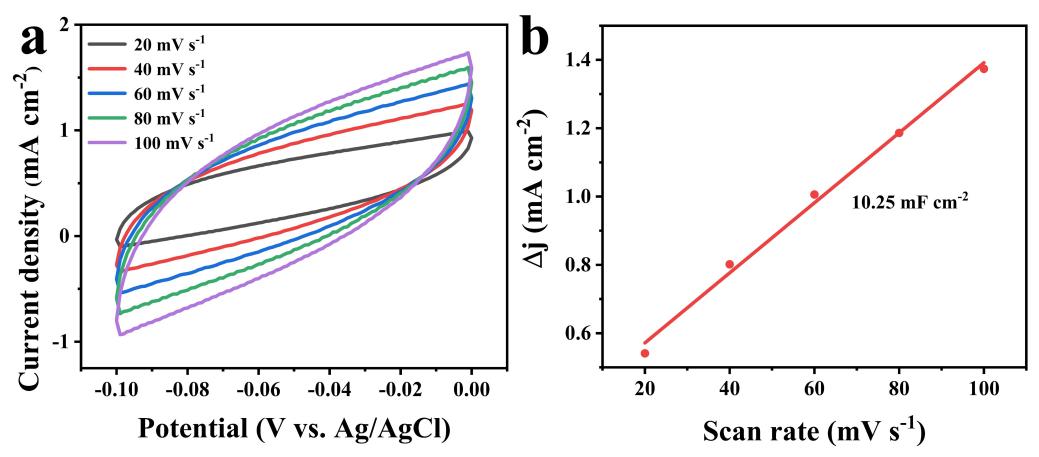


Figure S21 (a) CV curves of Mn-Co-Fe-P nanoarrays at different scan rates (20, 40, 60, 80 and 100 mV·s^-1^) in the non-faradaic potential region of -0.1 to 0 V vs. Ag/AgCl, (b) The capacitive current densities as a function of various scan rates for Mn-Co-Fe-P.


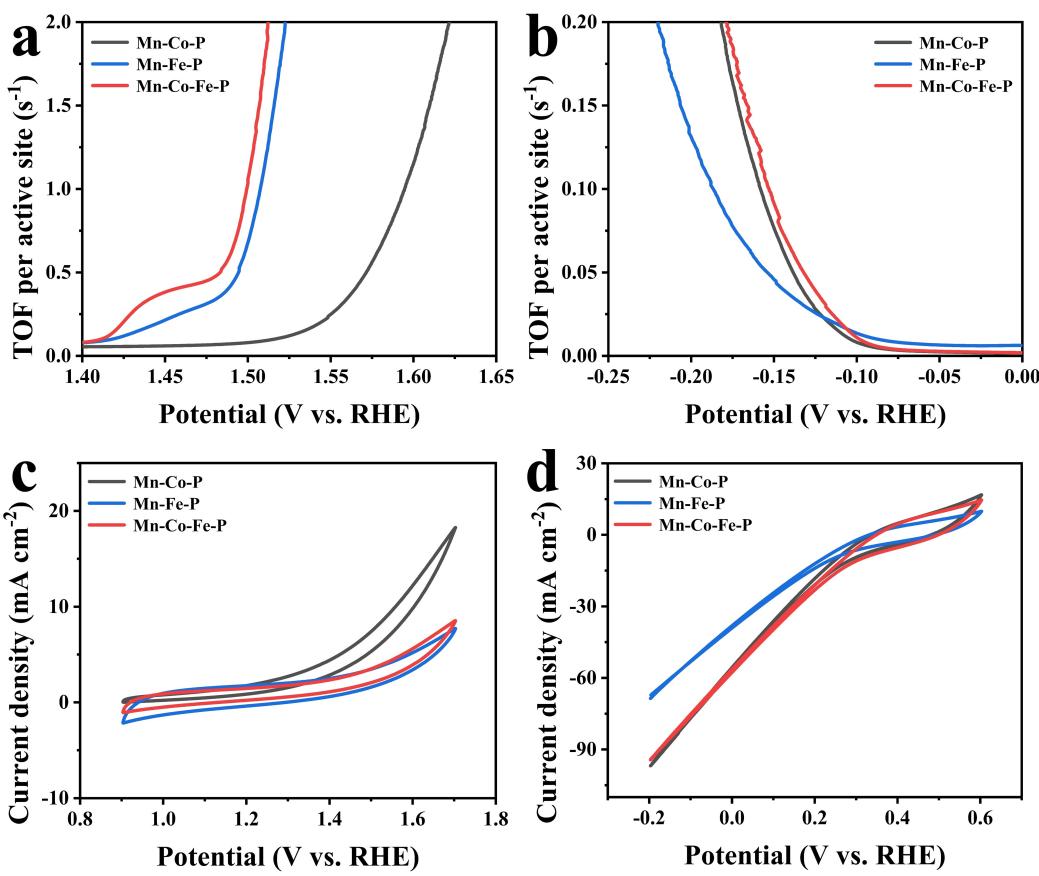


Figure S22 TOF curves of Mn-Co-Fe-P, Mn-Co-P and Mn-Fe-P nanoarrays for (a) OER and (b) HER; CV curves of Mn-Co-Fe-P, Mn-Co-P and Mn-Fe-P nanoarrays at a scan rate of 50 mV·s^-1^ in 1.0 M PBS (pH=7) for (c) OER and (d) HER.

**References**

[1] X. Hu, S. Zhang, J. Sun, L. Yu, X. Qian, R. Hu, Y. Wang, H. Zhao, J. Zhu, Nano Energy, **2019**, 56, 109-117.

[2] F.Q. Liu, J.W. Liu, Z. Gao, L. Wang, X.-Z. Fu, L.X. Yang, Y. Tao, W.H. Yin, F. Luo, Applied Catalysis B: Environmental, **2019**, 258, 117973.

[3] S. Liu, B. Liu, C. Gong, Z. Li, Appl Surf Sci, **2019**, 478, 615-622.

[4] Y. Pei, Y. Ge, H. Chu, W. Smith, P. Dong, P.M. Ajayan, M. Ye, J. Shen, Applied Catalysis B: Environmental, **2019**, 244, 583-593.

[5] C. Wang, H. Shang, Y. Wang, J. Li, S. Guo, J. Guo, Y. Du, Nanoscale, **2021**, 13, 7279-7284.

[6] P. Zhang, X.F. Lu, J. Nai, S.Q. Zang, X.W.D. Lou, Adv Sci (Weinh), **2019**, 6, 1900576.

[7] Z. Yi, C. Ye, M. Zhang, Y. Lu, Y. Liu, L. Zhang, K. Yan, Appl Surf Sci, **2019**, 480, 256-261.

[8] X.F. Lu, Y. Chen, S. Wang, S. Gao, X.W.D. Lou, Advanced Materials, **2019**, 31, e1902339.

[9] M. Wang, W. Fu, L. Du, Y. Wei, P. Rao, L. Wei, X. Zhao, Y. Wang, S. Sun, Appl Surf Sci, **2020**, 515, 146059.

[10] X. Xu, H. Liang, G. Tang, Y. Hong, Y. Xie, Z. Qi, B. Xu, Z. Wang, Nanoscale Advances, **2019**, 1, 177-183.

[11] H. Liao, X. Guo, Y. Hou, H. Liang, Z. Zhou, H. Yang, Small, **2020**, 16, e1905223.

[12] M. Lu, L. Li, D. Chen, J. Li, N.I. Klyui, W. Han, Electrochimica Acta, **2020**, 330, 135210.

[13] Y. Xing, D. Li, L. Li, H. Tong, D. Jiang, W. Shi, International Journal of Hydrogen Energy, **2021**, 46, 7989-8001.

[14] Y. Li, B. Jia, B. Chen, Q. Liu, M. Cai, Z. Xue, Y. Fan, H.P. Wang, C.Y. Su, G. Li, Dalton Transactions, **2018**, 47, 14679-14685.

[15] Y. Ge, J. Chen, H. Chu, P. Dong, S.R. Craig, P.M. Ajayan, M. Ye, J. Shen, ACS Sustainable Chemistry & Engineering, **2018**, 6, 15162-15169.

[16] B. Jiang, Z. Li, Journal of Solid State Chemistry, **2021**, 295, 121912.

[17] J. Liu, W. Li, Z. Cui, J. Li, F. Yang, L. Huang, C. Ma, M. Zeng, Chemical Communications (Camb), **2021**, 57, 2400-2403.

[18] D. Zhou, Z. Wang, X. Long, Y. An, H. Lin, Z. Xing, M. Ma, S. Yang, Journal of Materials Chemistry A, **2019**, 7, 22530-22538.

[19] M. Arif, G. Yasin, M. Shakeel, X. Fang, R. Gao, S. Ji, D. Yan, Chemistry an Asian Journal, **2018**, 13, 1045-1052.

[20] P. Wang, Y. Lin, L. Wan, B. Wang, Energy & Fuels, **2020**, 34, 10276-10281.

[21] G. Tang, Y. Zeng, B. Wei, H. Liang, J. Wu, P. Yao, Z. Wang, Energy Technology, **2019**, 7, 1900066.
